# Inositol Improves Cold Tolerance Through Inhibiting *CBL1* and Increasing Ca^2+^ Influx in Rapeseed (*Brassica napus* L.)

**DOI:** 10.3389/fpls.2022.775692

**Published:** 2022-03-17

**Authors:** Lei Yan, Liu Zeng, Ali Raza, Yan Lv, Xiaoyu Ding, Yong Cheng, Xiling Zou

**Affiliations:** Key Laboratory of Biology and Genetic Improvement of Oil Crops, Ministry of Agriculture, Oil Crops Research Institute of the Chinese Academy of Agricultural Sciences, Wuhan, China

**Keywords:** chilling stress, calcium ion, gene expression, transgenic plant, signaling pathways, stress tolerance

## Abstract

Rapeseed (*Brassica napus* L.) is an important oilseed crop worldwide. However, its productivity is significantly affected by various abiotic stresses, including cold stress. Among various stresses, cold stress is an important abiotic factor affecting plant growth, yield, and quality. The calcium channels are regarded as key pathways affecting cold tolerance in plants. Thus, improvement in cold tolerance is of great significance for crop improvement. The current study was designed to examine the beneficial role of exogenous inositol in improving cold stress tolerance in rapeseed. From the RNA-seq results, we identified 35 differently expressed genes encoding different inositol enzymes. The results show that inositol (a cyclic polyol) positively regulated cold tolerance by increasing calcium ion (Ca^2+^) influx in rapeseed. Furthermore, we found that the expression of calcineurin B-like (*CBL1*) gene was inhibited by inositol. On the other hand, overexpressed plant mediated the Ca^2+^ flux under cold stress suggesting the key role of inositol-Ca^2+^ pathway in cold tolerance. Moreover, the overexpression of *BnCBL1-2* in *Arabidopsis* represented that transgenic plants mediated the Ca^2+^ flux highlighting the vital role of the inositol-Ca^2+^ pathway in conferring cold stress. Our study provides new insights into rapeseed cold tolerance mechanism and introduces a feasible method to improve the cold tolerance of rapeseed quickly.

## Introduction

Cold stress, including chilling (>0°C) and freezing (<0°C) temperature, is one of the main abiotic factors affecting plant growth and productivity (Xin and Browse, 2000). In past years, the mechanism underlying plant cold tolerance has been extensively studied, and numerous components, such as transcription factors, amino acid metabolites, phosphatases, and protein kinases, have been identified in cold signaling pathways (Chinnusamy et al., 2003; Kou et al., 2018; Ding et al., 2019; Raza et al., 2021). Among them, the *CBF* (*C-repeat binding factors*)-*COR* (*cold-responsive*) signaling pathway is the most well-known. In this pathway, *CBFs* genes are rapidly induced by cold stress and then bind to the promoter regions of *COR* genes to activate their transcription (Chinnusamy et al., 2007; Shi et al., 2015; Jia et al., 2016; Zhao et al., 2016). The *COR* genes encode a series of osmolyte and cryoprotective proteins to protect the plant against cold stress (Ding et al., 2019). In addition, various hormones, including abscisic acid (ABA), brassinosteroids, ethylene, etc., play an important role in cold stress signaling pathways by regulating the expression level of stress-responsive genes (Chen and Gusta, 1983; Huang et al., 2017; Tan et al., 2017; Rubio et al., 2019).

In addition to CBF-COR signaling and hormone-induced pathways, calcium ions (Ca^2+^) also play an essential role in regulating plant cold response. Cytosolic Ca^2+^ are rapidly increased by cold stress, which is considered as one of the earliest cold signaling events in plants (Knight et al., 1996; Ding et al., 2019). The plasma membrane and endoplasmic reticulum-localized G protein regulator *COLD1* (CHILLING TOLERANCE DIVERGENCE1) coupled with *RGA1* (RICE G PROTEIN α SUBUNIT1) were found to improve the cold tolerance by regulating the influx of intracellular Ca^2+^ in rice (Ma et al., 2015). Some studies also indicated that Ca^2+^ might act as an important secondary messenger mediating low-temperature sensing in plants. For instance, CDPKs (Ca^2+^-dependent protein kinases), CBLs (calcineurin B-like), CIPKs (CBL-interacting protein kinases), and ANNEXIN1 (Ca^2+^ permeable transporter) were shown to modulate the cold tolerance in plants (Cheong et al., 2003; Abbasi et al., 2004; Komatsu et al., 2007; Weckwerth et al., 2015; Li et al., 2019; Liu et al., 2020). Inositol is a cyclic polyol that participates in numerous physiological processes, including signal transduction and stress adaptation in plants (Loewus and Murthy, 2000). A recent study shows that the overexpression of the rice inositol-coding gene (*OsIMP*) increases the accumulation of inositol and cold tolerance in tobacco by regulating the antioxidant enzyme activities (Zhang et al., 2017). However, the inositol-induced cold stress tolerance mechanism is yet to be reported.

Rapeseed (*Brassica napus* L.) is an important overwintering oilseed crop globally. It is mainly grown in the Yangtze River Basin of China, Canada, Central, and Northern Europe. Nevertheless, its yield parameters have been significantly altered due to the increased exposure to cold stress (Huang et al., 2021; Raza, 2021). In January 2008, south China suffered severe freezing weather, which reduces the rapeseed yield by 10.9% (Zhang et al., 2008). Therefore, there is a dire need to examine the molecular and biochemical mechanisms to enhance rapeseed production. Over the past few years, several attempts have been made to identify the col-responsive mechanisms in rapeseed. For instance, Zeng et al. (2018) identified eight conserved, and two novels differentially expressed miRNAs that positively regulate cold stress tolerance in rapeseed. Previous studies showed six cold-tolerant rapeseeds cultivars and C18 had a higher ability to regulate hormonal, osmotic, and antioxidative activity along with gene transcriptional level than in cold-sensitive cultivar (C20) under cold stress (Yan et al., 2018, 2019). Only a few cold-responsive genes have been identified in rapeseed, and most of them are homologous to *CBF* or *COR,* including *BnCBF5, BnCBF17,* and *BnCOR25* (Savitch et al., 2005; Chen et al., 2011).

In this study, the comparison of transcriptomes was performed between cold-tolerant (C18) and cold-sensitive (C20) cultivars. The transcriptome results indicated that inositol was involved in the cold response. Therefore, rapeseed seedlings were treated with exogenous inositol to identify the possible mechanism of inositol-mediated cold tolerance. The results showed that inositol increased the expression of *CORs* genes independent of *CBF* or ABA and improved cold tolerance of rapeseed by increasing Ca^2+^ influx. In addition, we revealed that a feedback regulatory pathway composed of inositol, *CBL1*, and Ca^2+^ playing a key role in cold-induced cytoplasmic calcium accumulation and cold tolerance in rapeseed.

## Materials and Methods

### Plant Materials, Freezing Stress, and RNA Isolation

The seeds of C18 (cold-tolerant) and C20 (cold-sensitive) cultivars were provided by the Oil Crops Research Institute, Chinese Academy of Agricultural Sciences (Wuhan, China). Rapeseed seedlings were grown in small pots having soil in a growth chamber with a 16-/8-h light/dark cycle at 22°C. Then, the 21-day-old seedlings (three-leaf stage) were placed in low-temperature incubators at 4°C for 24 h. Cold stress was imposed at a temperature of 2°C for 1 h, and then the temperature was reduced gradually to 0°C, −2°C for 1 h, respectively. The leaf samples from all the seedlings grew under corresponding controls (22°C when sampled under 2°C, 0°C and −2°C), 2°C, 0°C and −2°C were collected, frozen in liquid nitrogen, and stored at −80°C for RNA extraction and further use. *Arabidopsis* freezing treatment was carried out as described by Zhao et al. (2016). RNA extraction and purification were performed as described in Yan et al. (2019). Using the above-mentioned samples, the RNA-seq was performed using the Illumina Hiseq 2000 (LC, Science, and Houston, TX) platform, and then the raw reads were generated. The whole experiment was carried out with three biological replications.

### Analysis of RNA Sequencing Data

Raw data were processed with Fast QC 10.1 software and filtered by removing the unqualified reads. The descriptive statistics for the clean data, such as Q20, Q30, and GC content, were calculated. The clean reads of each library were mapped to the *Brassica napus* L. genome using Hisat 2.0 software to count the mapping rate.^1^ The remaining sequences were considered as clean reads (the corresponding data have been submitted into the database of National Center for Biotechnology Information, Short Read Archive (NCBI, SRV) with accession number PRJNA562064). Fragment per kilobase of exon per million fragments mapped (FPKM) value was used to evaluate the gene expression level.

### Identification and Analysis of DEGs

The DEGseq R package was used to identify differentially expression genes (DEGs). The integration of a *p* < 0.05 and an absolute value of log2 ratio > 1 were used to identify DEGs. Gene ontology (GO)^2^ and MapMan^3^ analysis were performed for the functional analysis of DEGs.

### qRT-PCR Analysis

The above-mentioned RNA samples were used for qRT-PCR analysis. The primers were designed using Primer 5.0 software and listed in Supplementary Table S5. The *Brassica napus* actin gene-specific primer (GeneBank: AF111812) was used as a control to normalize the expression data (Wang et al., 2014). The qRT-PCR was carried out with the TransStart Tip Green qPCR SuperMix (TransGen, China) on StepOnePlus real-time PCR system (Applied Biosystems, United States). The condition for PCR was as follows: 95°C for 30s and 40 cycles of 95°C for 10s, followed by 60°C for 30s (extension and signal acquisition). The relative expression levels were calculated using the 2^−∆∆Ct^ method.

### Measurement of Endogenous Inositol and ABA Concentrations

Endogenous inositol and ABA concentration were estimated using the ELISA kit produced by You Xuan Biological Technology Co. Ltd. (Shanghai, China). A total of 0.1 g of leaves was homogenized in chilled pestle and mortar using the 50 mmol L^−1^ phosphate buffer (pH 7.4). The homogenate was centrifuged at 4,000 rpm for 10 min. Then, the supernatant was collected into another tube to measure the inositol concentration following the kit manufacturer’s instructions.

### Application of Exogenous Inositol

The 21-day-old seedlings of four cold-sensitive cultivars (Zheyou21, Ningyou18, C06, and C20) were pretreated with different concentrations (0.1, 0.5, 1.0, 2.0, 5.0, and 8.0 g L^−1^) of inositol solution, and the CK plants were treated with water. The seedlings were divided into two groups. In the first group, the seedlings were treated with freezing stress (−2°C) immediately, and the second group seedlings were treated with freezing stress (−2°C) 2 days after the pretreatment with inositol. After the freezing treatment, the survival rate was evaluated as described in our previous report (Yan et al., 2018).

### Ca^2+^ Flux

Net Ca^2+^ fluxes of leaves of 21-day-old seedlings were measured at the YoungerUSA Xuyue (Beijing) BioFunction Institute by using Non-invasive Micro-test Technology (NMT100 Series①, YoungerUSA LLC, Amherst, MA 01002, United States) Xuyue (Beijing) Sci. & Tech. Co., Ltd., Beijing, China, and imFluxes V2.0 (YoungerUSA LLC, Amherst, MA 01002, United States) Software (Ma et al., 2015).

### Cloning of *CBL1-2* and Identification of Transgenic Plants

The CDS of *BnCBL1-2* was cloned by primers 5′- ATGGGCTGCTTCCACTCAA-3′ and 5′-TCATGTGGCAATCTCATCGAC-3′ and inserted into binary vector pBCXUN (ubiquitin promoter) linearized with *Sma* I and *BamH* I enzymes. The recombinant plasmid pBCXUN-BnCBL1-2 (Supplementary Figure S5) was introduced into Agrobacterium tumefaciens strain GV3101 using the heat shock method. Agrobacterium-mediated transformation of *Arabidopsis* was carried out as described by Clough and Bent (1998).

To check the effect of exogenous inositol on cold-responsive markers gene (*CBFs*, and *CORs*) and ABA contents, the 21-day-old seedlings of C20 were sprayed with 0.5 g L^−1^ of inositol solution water (control). Then, the seedlings were divided into two groups. One group continued to grow under 22°C, and the other was treated at 4°C for 1 h. After 1 h, green leaves were harvested and were stored at −80°C for the qRT-PCR.

Similarly, the 21-day-old seedlings of C20 were used to evaluate the transient effect of exogenous inositol on *CBLs* genes. The seedlings were subjected to the cold stress as 4°C for 1, 2, 4, 8, 12, and 24 h, 2°C for 1 h, 0°C for 1 h, and −2°C for 1 h. Green leaves were harvested at each time point and were stored at −80°C for the qRT-PCR. Meantime, the seedlings with the pretreatment of inositol and water under 22°C were also harvested as the CK. For durative effect, the cold stress was imposed after cold acclimation of 4°C for 24 h to 21-day-old seedlings of C20. The cold stress treatment was set as 2°C for 1 h, 0°C for 1 h, and −2°C for 1 h. The pretreatment of inositol and the harvest of samples were the same as the explained earlier. The primers used for qRT-PCR are shown in Supplementary Table S5.

### Statistical Data Analysis

Statistical analysis were performed using SPSS statistical package (SPSS Student version 15.0). Significant differences were evaluated using the two-tailed Student’s t-test or one-way ANOVA and Duncan’s test. All test differences at *p* ≤ 0.05 were considered to be significant. All the error bars were SD (Standard Deviation) value.

## Results

### Inositol Phosphate Pathway Had Been Enriched and Displayed Differences Between C18 and C20

C18 and C20 were verified to be cold-tolerant and cold-sensitive cultivars in previous morphological and physiological experiments (Yan et al., 2018, 2019). To identify the molecular mechanism of cold tolerance, the two cultivars were exposed to 2°C, 0°C, −2°C, and 22°C (CK, control) for cold stress treatment and 36 cDNA libraries (two genotypes × six treatments × three biological replicates) were constructed for RNA-seq (Supplementary Figure S1). After a series of processing, such as filtering and quality control (Supplementary Table S1), the sequencing data were used for further analysis. With the criterion of |log_2_FC| > 1 and q value < 0.05, a total of 31,392 DEGs were identified between cold stress and corresponding control.

**Figure 1:**
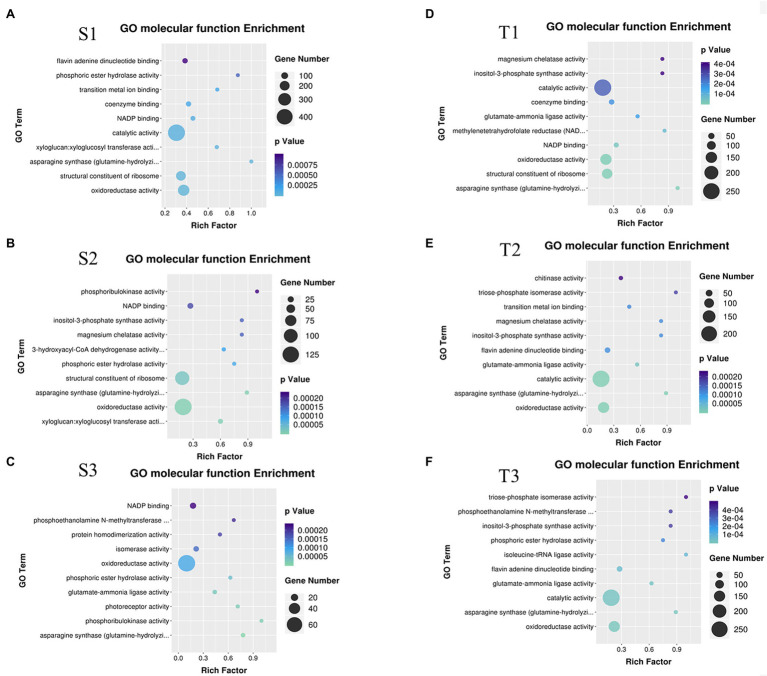
Gene ontology (GO) molecular function enrichment analysis of DEGs. **(A)** GO molecular function enrichment for S1 (the sensitive variety C20 under 2°C compared to corresponding control); **(B)** GO molecular function enrichment for S2 (C20 under 0°C compared to corresponding control); **(C)** GO molecular function enrichment for S3 (C20 under −2°C compared to corresponding control); **(D)** GO molecular function enrichment for T1 (the tolerant variety C18 under 2°C compared to corresponding control); **(E)** GO molecular function enrichment for T2 (C18 under 0°C compared to corresponding control); **(F)** GO molecular function enrichment for T3 (C18 under −2°C compared to corresponding control).

**Figure 2:**
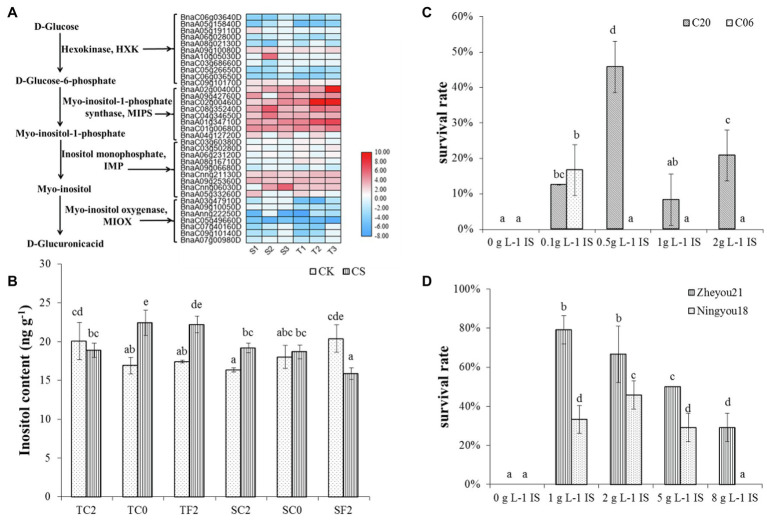
Inositol involved in cold tolerance in rapeseed. **(A)** The expression analysis of DEGs involved inositol biosynthesis and degradation. Heat map data is log2 (Fold change). T1: the tolerant variety C18 under 2°C compared to corresponding control; T2: C18 under 0°C compared to corresponding control; T3: C18 under −2°C compared to corresponding control; S1: the sensitive variety C20 under 2°C compared to corresponding control; S2: C20 under 0°C compared to corresponding control; S3: C20 under −2°C compared to corresponding control. **(B)** Endogenous inositol concentrations under different cold treatment. TC2: C18 under 2°C; TC0: C18 under 0°C; TF2: C18 under −2°C; SC2: C20 under 2°C; SC0: C20 under 0°C; SF2: C20 under −2°C; CS: cold stress; CK: 22°C. Values were means of three replications, and the bars were represented the standard error of means. Different letters were represented significant differences according to Duncan’s multiple range test (*p* < 0.05). **(C,D)** Exogenous inositol improved the cold tolerance of rapeseed. IS: inositol.

Further, these DEGs were subjected to GO enrichment. The results of GO molecular function enrichment showed oxidoreductase activity, asparagine synthase (glutamine-hydrolyzing) activity, NADP binding, and phosphoric ester hydrolase activity were enriched in S1, S2, S3 (cold-sensitive cultivar C20 under 2°C, 0°C, −2°C compared to 22°C); oxidoreductase activity, asparagine synthase (glutamine-hydrolyzing) activity, catalytic activity, glutamate–ammonia ligase activity, and inositol-3-phosphate synthase activity were enriched in T1, T2, T3 (cold-tolerant cultivar C18 under 2°C, 0°C, −2°C compared to 22°C; Figure 1). The functional enrichment analysis by Mapman indicated that the pathway of inositol phosphate was enriched (Supplementary Figure S2). Inositol biosynthesis and degradation were enriched under cold stress, and 35 related DEGs were identified to be differentially expressed (Figure 2A). Of these 35 DEGs, 28 DEGs are involved in inositol biosynthesis, encoding three enzymes (hexokinase HXK, myo-inositol-1-phosphate synthase MIPS, and inositol monophosphate IMPase), and seven DEGs involved in inositol degradation, encoding myo-inositol oxygenase (MIOX; Figure 2A). Among these 35 DEGs, 14 DEGs were randomly selected for qRT-PCR-based validation. The expression patterns for these DEGs were generally consistent between RNA-seq and qRT-PCR data (Supplementary Figure S3), indicating the reproducibility of the RNA-seq data.

Among different *HXK* genes, two *HXK2* genes were upregulated in response to cold stress in both cultivars, and one *HXK3* and *HXK4* were upregulated in C20. Seven *HXK3* were downregulated in both cultivars (C18 and C20; Figure 2A). MIPS catalyzes the conversion of glucose 6-phosphate to myo-inositol 1-phosphate, which is a rate-limiting step of the inositol biosynthetic pathway in plants. All the eight DEGs encoding MIPS were increased in both cultivars under cold stress; however, the genes in C18 had a stronger increasing trend than that in C20. For IMPase, eight upregulated DEGs were identified in C18 under 2°C, while there were only four upregulated genes identified in C20 (Figure 2A). Notably, the expression trends have varied under 0°C and −2°C. For instance, more *MIOXs* genes were downregulated in C18 than C20 under cold stress. Especially under 0°C, seven downregulated *MIOXs* were identified in C18, and only three downregulated MIOXs were found in C20. The different expression patterns of genes responsible for myo-inositol biosynthesis and degradation between C18 and C20 might result in higher endogenous inositol accumulation in C18 than in C20 (Figure 2A).

### Endogenous Inositol Content Increased in C18 and Decreased in C20 With Varying Temperature

The endogenous inositol concentration in C18 was 18.9 ng g^−1^ and 20.1 ng g^−1^ under 2°C and CK, and there was no significant difference. With the continually dropping temperature, the inositol content in C18 increased significantly (22.4 and 22.2 ng g^−1^ under 0°C and −2°C treatment) compared to control conditions (16.9 and 17.4 ng g^−1^; Figure 2B). The inositol content of C20 under 2°C (19.2 ng g^−1^) was higher than that under CK (16.4 ng g^−1^). Notably, at 0°C, there were no significant differences (18.7 and 18.0 ng g^−1^). However, the inositol content (15.9 ng g^−1^) of C20 under −2°C was decreased compared to control (20.4 ng g^−1^; Figure 2B). In a nutshell, the results showed that endogenous inositol concentration increased in C18 and decreased in C20 with the gradually dropping temperature.

### Exogenous Application of Inositol Improved Cold Tolerance In Rapeseed

To investigate the effect of inositol on cold tolerance in rapeseed, four representative sensitive cultivars (Zheyou21, Ningyou18, C06, and C20; Yan et al., 2018) were chosen to be treated with exogenous inositol under cold stress. The results presented that all the sensitive varieties with inositol pretreatment were found to be more cold-tolerant than CK (Figures 2C,D). Interestingly, control seedlings were failed to survive under −2°C. At the same time, the treated cultivars showed better performed. The survival rate of Zheyou21, Ningyou18, C06, and C20 was 79, 46, 17, and 46%, respectively, when pretreated with optimum concentration of inositol 1.0 g L^−1^, 2.0 g L^−1^, 0.1 g L^−1^, and 0.5 g L^−1^, respectively (Figures 2C,D).

### Exogenous Inositol Application Increased the Expression of *CORs* Gene *via* a Pathway Independent of CBF or ABA

**Figure 3:**
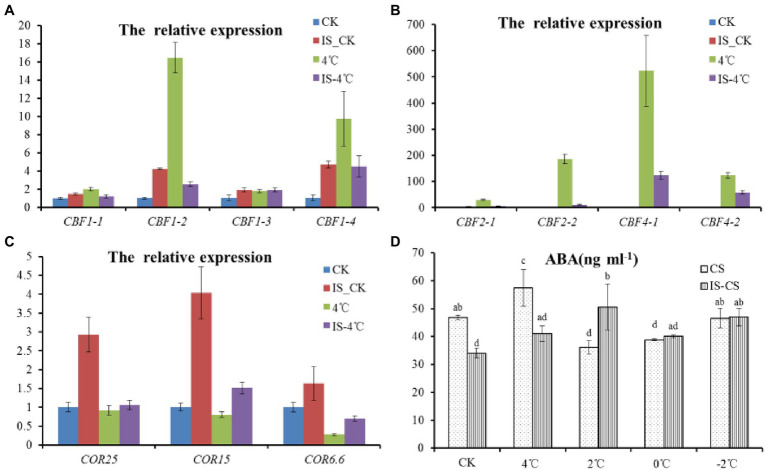
Expression patterns of *CBFs*, *CORs*, and ABA content with exogenous inositol under cold stress. **(A)** The expression patterns of *CBF1* (*CBF1-1- CBF1-4*) with exogenous inositol under cold stress; **(B)** The expression patterns of *CBF2* (*CBF2-1* and *CBF2-2*) and *CBF4* (*CBF4-1* and *CBF4-2*) with exogenous inositol under cold stress; **(C)** The expression patterns of *CORs* (*COR25*, *COR15*, and *COR6.6*) with exogenous inositol under cold stress; **(D)** ABA content change with exogenous inositol under cold stress. CK: 22°C; 4°C: 4°C treatment for 1 h; IS_CK: 22°C for 1 h with 0.5 g L^−1^ of exogenous inositol; IS_4°C: 4°C for 1 h with 0.5 g L^−1^ of exogenous inositol.

To clarify signal transduction of inositol, the relation was explored between inositol and known signaling pathways involved in cold stress, such as the CBF pathway. Therefore, the expression of several markers genes, including *CBFs* and *CORs* were analyzed after exogenous inositol pretreatment. Under 22°C, the expression of *BnCBF1*, *BnCBF2*, and *BnCBF4* was slightly increased after exogenous inositol application (Figures 3A,B). However, at 4°C, the expression of *BnCBF1*, *BnCBF2*, and *BnCBF4* was decreased dramatically compared with CK. The result showed that exogenous inositol increased cold tolerance, not by increasing the expression of *CBFs*. Besides, the expression pattern of *CORs* after exogenous inositol application was also analyzed. Under 22°C, the expressions of *BnCOR6.6*, *BnCOR15*, and *BnCOR25* increased significantly after exogenous inositol application (Figure 3C). At 4°C, the expression level of *BnCOR6.6* and *BnCOR15* was increased in inositol-treated seedlings than CK. However, for the expression of *BnCOR25*, there was no significant difference between the inositol treatment and CK.

Considering the expression of *BnCOR25* is regulated by ABA (Chen et al., 2011), the ABA concentration of seedlings after exogenous inositol application was detected. The results showed that, under 0°C and −2°C, there was no significant difference for ABA concentration between inositol treatment and CK (Figure 3D). In addition, the genes involved in CBF or ABA pathway were explored in the transcriptome data, and there was no difference of expression between C18 and C20 cultivars under cold stress (Supplementary Table S2). All of the above evidence indicated that exogenous inositol increased cold tolerance, not through the ABA pathway, but maybe by regulating the expression levels of cold-responsive marker genes.

### Inositol Increased Ca^2+^ Influx Under Cold Stress

**Figure 4:**
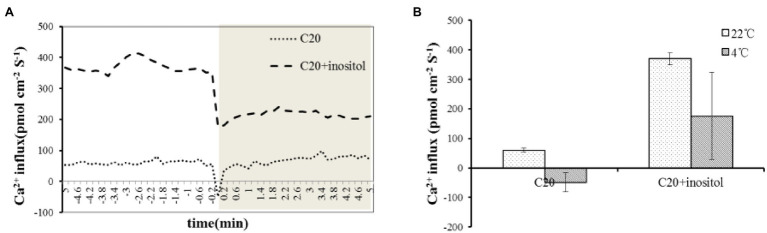
Ca^2+^ influx upon Cold Shock in C20 spayed water and inositol. **(A)** NMT measurements show extracellular Ca^2+^ influx upon on cold shock in live leaves of C20 spayed with water and inositol (*n* = 3). **(B)** The mean Ca^2+^ fluxes in 22°C and 4°C.

After excluding the CBF and ABA pathway, it is speculated that the mechanism of inositol improving cold tolerance may be related to Ca^2+^ flux. Therefore the Ca^2+^ flux value in C20 seedlings treated with water and inositol was evaluated. Under 22°C, the initial Ca^2+^ flux value of inositol and water treated seedlings was 370.1 and 60.3 pmol cm^−2^ s^−1^, respectively (the positive value represents Ca^2+^ efflux, and a negative value represents Ca^2+^ influx). Under 4°C, the Ca^2+^ influx of inositol and water treated seedlings increased by 195.5 and 108.7 pmol cm^−2^ s^−1^, respectively, and the Ca^2+^ flux value finally reached 175.6 and − 48.4 pmol cm^−2^ s^−1^, respectively (Figures 4A,B). Compared with the control, inositol treatment led to a stronger calcium influx. The results suggested that inositol increased Ca^2+^ influx under cold stress, resulting in enhanced cold tolerance in rapeseed.

### Inositol Inhibited *BnCBL1-2* Under Cold Stress

In our study, inositol was proved to increase Ca^2+^ influx under cold stress which resulted in enhanced cold tolerance in rapeseed. In other studies, the combination of different concentrations of inositol and Ca^2+^ significantly promoted the growth of Chinese cabbage and pepper seedlings (Liu et al., 2017; Yang et al., 2017).

**Figure 5:**
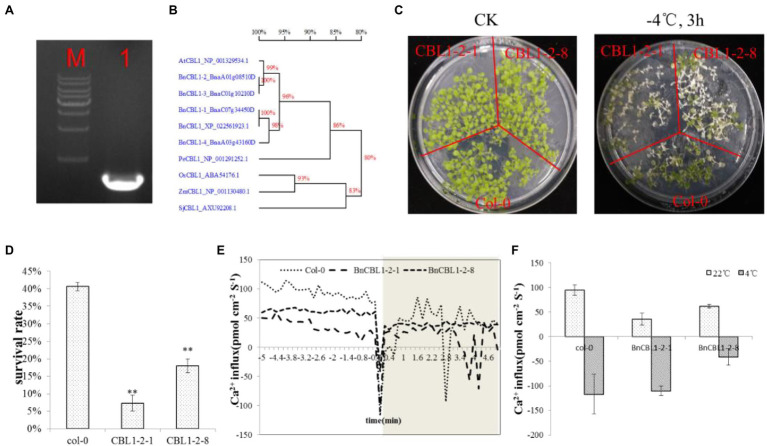
*BnCBL1-2* reduced cold tolerance and Ca^2+^ flux. **(A)** Cloning of *BnCBL1-2*, M: 1 Kb Maker, 1: 642 bp; **(B)** Phylogenetic relationships of *CBL1* genes in plants; **(C)** The phenotype of Col-0 and *BnCBL1-2*-overexpressing *Arabidopsis* lines under 22°C and − 4°Ctreatment; **(D)** The survival rate of Col-0, *CBL1-2-1*, and *CBL1-2-8* after 3 h of −4°C freezing stress; **(E,F)** Ca^2+^ influx under Cold Shock in col-0 and overexpressing *BnCBL1-2* Arabidopsis. **(E)** NMT measurements show extracellular Ca^2+^ influx upon on cold shock in live leaves of col-0 and overexpressing *BnCBL1-2* Arabidopsis (*n* = 3). **(F)** the mean Ca^2+^ fluxes in 22°C and 4°C.

Then, we identified all the Ca^2+^ related gens in both the transcriptome of C18 and C20, and 351 DEGs were obtained to be different expressed in C18 and C20 (Supplementary Table S3). It has also been reported that some calcium sensors, such as *CBL1*, act as a rate-limiting factor to response to stress signals (Cheong et al., 2003). Of these 351 DEGs, there were four *CBL1s*. The expression of all the four BnCBL1s was increased in C20 under cold stress. There were no significant differences of expressions of *BnCBL1-3* and *BnCBL1-4* in C18 under cold stress. The expressions of *BnCBL1-1* and *BnCBL1-2* were decreased in C18 under cold stress (Supplementary Table S4). The result indicated that *BnCBL1* might be involved in signaling in cold tolerance. With the pretreatment of exogenous inositol, the expression of *BnCBL1-2* decreased in C20 under freezing stress (Supplementary Figure S4). *BnCBL1-2* was overexpressed in *Arabidopsis*, and two lines, *BnCBL1-2-1* and *BnCBL1-2-8*, were selected. After the cold treatment at −4°C for 3 h, approximately 41% of WT and 7–18% of *CBL1*-overexpressing plants recovered from the freezing treatment The survival rate of transgenic lines was significantly lower than WT plants (Figures 5C,D). The results indicated that overexpression of *BnCBL1-2* reduced cold tolerance.

**Figure 6:**
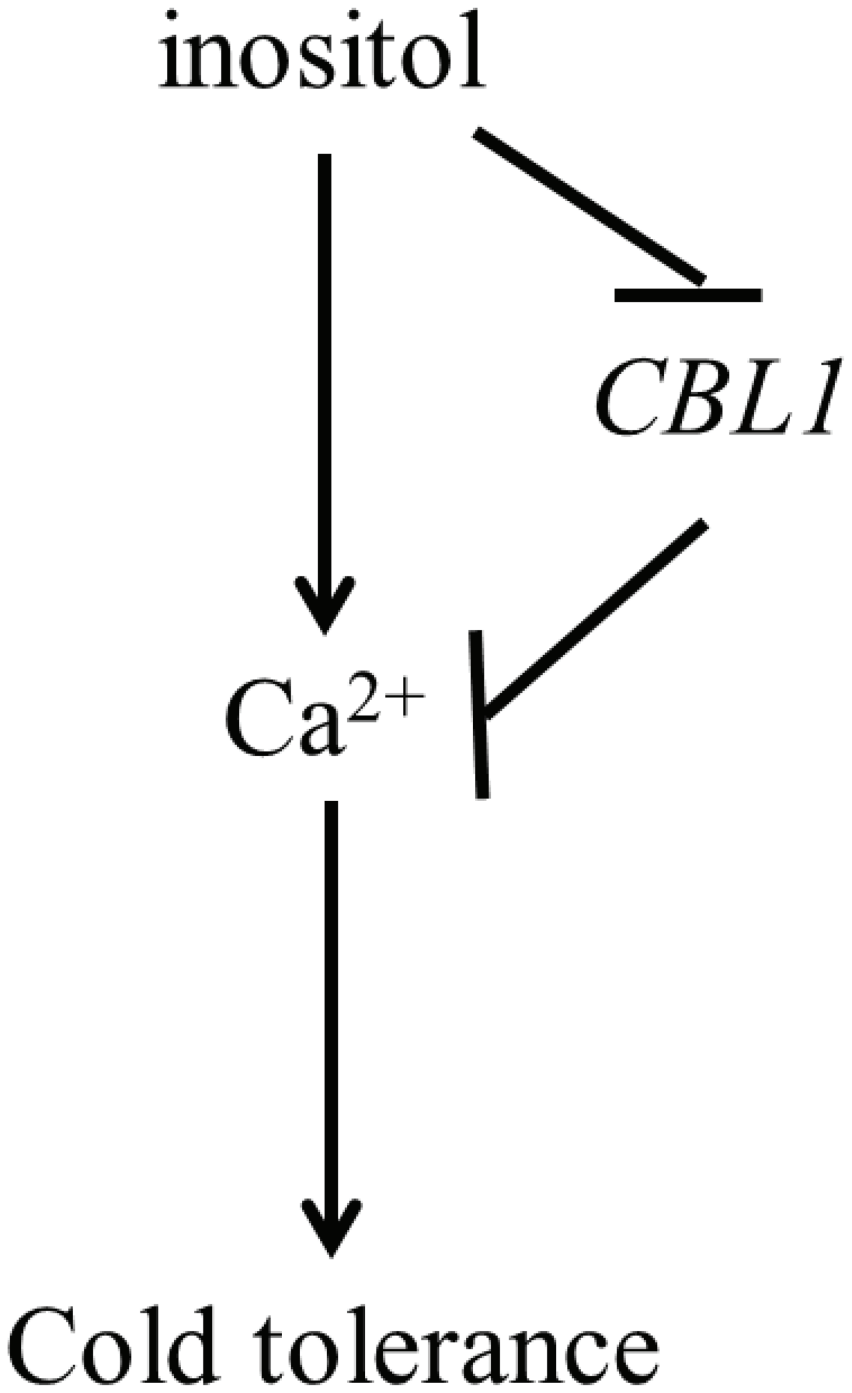
The model of pathway of inositol, CBL1 and Ca^2+^ response to cold stress. Arrows were represented positive regulation, whereas lines ending with a bar showed negative regulation.

The further experiment showed that overexpression of *BnCBL1-2* reduced cold tolerance and Ca^2+^ flux (Figures 5E,F). This research also revealed that inositol inhibited the expression of *BnCBL1-2* (*BnaA01g08510D*) under cold stress. Therefore, it is proposed there is a mechanism that inositol regulates the Ca^2+^ signaling and the expression of *CBL1* (Figure 6), which is a brand-new signal pathway under cold stress in plants.

### Overexpressing *BnCBL1-2* Weaken the Ability of Ca^2+^ Influx Regulation in Plants

The Ca^2+^ flux was measured in WT and transgenic *Arabidopsis* with overexpressing *BnCBL1-2*. Under normal condition (22°C), in mesophyll cells, the initial Ca^2+^ flux values were 94.7, 35.6, and 61.7 pmol cm^−2^ s^−1^, respectively in WT, and overexpressing (*BnCBL1-2\u20131* and *BnCBL1-2\u20138*) transgenic plants. Under cold stress (4°C), the Ca^2+^ flux of WT, overexpressing (*BnCBL1-2\u20131* and *BnCBL1-2\u20138*) plants in mesophyll cells were − 117.2, −101.8, and − 41.1 pmol cm^−2^ s^−1^, respectively (Figures 5E,F). Compared with overexpressing *BnCBL1-2* plants, WT plants showed more Ca^2+^ efflux under normal conditions and more Ca^2+^ influx under cold stress, suggesting a stronger ability in Ca^2+^ regulation.

## Discussion

### Inositol Improved Cold Tolerance in Rapeseed

The best understood cold signaling pathway is the *ICE-CBF* transcriptional cascade, and the *CBF* genes play crucial roles in this cascade. In this study, three *CBF* related DEGs, *BnaA03g13620D* (homologous to *AtCBF1*), *BnaAnng34260D* (homologous to *AtCBF1*), and *BnaA10g07630D* (homologous to *AtCBF4*) were identified (Supplementary Table S2). The *BnCBF4* (*BnaA10g07630D*) was overexpressed in *Arabidopsis*, and the plants had a retarded flowering period (data not shown), which was similar to the previous studies (Jaglo-Ottosen et al., 1998; Gilmour et al., 2004). Overexpression of *CBFs* not only improved freezing tolerance but also led to dwarfism and late flowering (Jaglo-Ottosen et al., 1998; Gilmour et al., 2004). Therefore, the *CBFs* are not ideal target genes in transgenic breeding due to their negative effects. Previous transcriptome analysis showed that only 12% of the cold-responsive genes were controlled by *CBFs* (Fowler and Thomashow, 2002), which indicated there were other unexplored important pathways responding to cold stress.

Inositol, a cyclic polyol, is involved in various physiological processes and participates in programmed cell death, pathogen resistance, and stress adaptation in plants (Bohnert et al., 1995; Chaouch and Noctor, 2010; Bruggeman et al., 2014). In *Medicago falcata*, the concentration of inositol increased significantly under low temperature, and the inositol concentration in *Medicago falcata* (cold-tolerant) was higher than *Medicago sativa* (cold-sensitive) at 5°C (Tan et al., 2013). In animals, inositol was also reported to be involved in cold tolerance. For instance, the inositol concentrations increased up to 400-fold and peaked at 147 nmol mg^−1^ fresh mass in overwintering flies during the winter (Vesala et al., 2012). Previous investigations also reported that inositol alleviated the damage of cold stress on maize and rice seedlings (You et al., 2000; Guo et al., 2014; Yao and Xia, 2014). When inositol was used as a seed coating, it significantly increased the germination rate and fresh weight in maize under cold stress (Yu et al., 2009). Notably, the combination of different concentrations of inositol and Ca^2+^ significantly promoted the growth of Chinese cabbage and pepper seedlings (Liu et al., 2017; Yang et al., 2017). In this study, inositol-3-phosphate synthase activity was enriched in T1, T2, T3, and S2 (Figure 1), and C18 showed higher inositol biosynthesis gene expression and lower degradation gene expression than in C20 (Figure 2A). Further, the endogenous inositol concentration was increased in C18, and decreased in C20 with the continually dropping temperature (Figure 2B), suggesting the crucial role of inositol in cold tolerance. Besides, exogenous application of inositol increases cold tolerance of all four sensitive rapeseed cultivars (Figures 2C,D). Briefly, these results indicated that inositol played an important role in cold tolerance in rapeseed.

### A New Signaling Pathway of Inositol Regulated Ca^2+^ and *CBL1* Modulates Cold Tolerance

As a secondary messenger, Ca^2+^ are participate in many biological processes and plays a critical role in signaling pathways under stress conditions in plants. Under stress, stress signals typically boost the Ca^2+^ levels over the threshold and activate calcium sensors. In this study, inositol positively regulated cold tolerance by increasing Ca^2+^ influx in rapeseed. Inositol is an important precursor of inositol phospholipids (Downes and Macphee, 1990). Phosphatidylinositol (4, 5) P2 are hydrolyzed by phospholipase C (PLC) to InsP3 and diacylglycerol (DAG). In animal cells, InsP3 activate InsP3 receptor (Ca^2+^ channel) and release Ca^2+^ (Valluru and Van den Ende, 2011). These outcomes suggest that there is a crosstalk between the signal pathway of inositol and Ca^2+^ in improving cold tress tolerance.

Some calcium sensors, such as *CBL1*, act as a rate-limiting factor in response to stress signals (Cheong et al., 2003). In *Arabidopsis*, *CBL1* improved the salt and drought stress tolerance but decreased cold stress tolerance (Cheong et al., 2003). Overexpression of *BnCBL1* improved tolerance to high salinity and low phosphate conditions in rapeseed (Chen et al., 2012). In this study, inositol increased Ca^2+^ influx under cold stress (Figures 4A,B), 351 Ca^2+^ DEGs and found *CBL1s* had different expression trend in both cultivars. Among different *CBL1s*, there were no significant differences in the expression levels of *BnCBL1-3* and *BnCBL1-4*. On the other hand, the expression levels of *BnCBL1-1* and *BnCBL1-2* were decreased in C18 under cold stress, whereas the expressions of four *BnCBL1s* increased in C20 under cold stress (Supplementary Table S4). Moreover, exogenous inositol decreases the expression of *BnCBL1-2* in C20 under freezing stress (Supplementary Figure S4). Overexpression of *BnCBL1-2* reduced cold tolerance and Ca^2+^ flux (Figures 5E,F). It was assumed that overexpression of *BnCBL1-2* may disturb the Ca^2+^ homeostasis in plants, leading to the failure in sending cold signals to the downstream signaling pathway. This research also revealed that inositol inhibited the expression of *BnCBL1-2* (*BnaA01g08510D*) under cold stress. Therefore, it is proposed there is a mechanism that inositol regulates the Ca^2+^ signaling and the expression of *CBL1* (Figure 6), which is a brand-new signal pathway under cold stress in plants.

## Conclusion

Rapeseed is considered one of the most important and economical oilseed crops around the world. However, cold stress significantly affects its growth and production in China. Therefore, the current study identified an inositol-mediated mechanism that helps in improving cold stress tolerance in rapeseed seedlings. Briefly, our results show that inositol positively regulated the cold tolerance by increasing Ca^2+^ influx in rapeseed. Inositol also inhibited the expression of the *CBL1* gene under stress conditions. Besides, overexpressed *Arabidopsis* plants mediated the Ca^2+^ flux under cold stress suggesting the key role of the inositol-Ca^2+^ pathway in cold tolerance in rapeseed. Therefore, future works should focus on how inositol interacts and regulates the expression of cold-responsive marker genes that help mitigate the adverse effect of cold stress on plants.

## Data Availability Statement

The original contributions presented in the study are publicly available in NCBI under accession number PRJNA562064.

## Author Contributions

LY conducted the experiment, analyzed the data, and wrote the paper. LY and AR wrote and revised the manuscript. LZ and XD provided the reagents and materials. YL, YC, and XZ helped in the relevant literature. XZ supervised the study, designed the experiment, and revised the paper. All authors contributed to the article and approved the submitted version.

## Funding

This work was supported by the National Key Research and Development Program (2017YFD0101700), Agricultural Science and Technology Innovation Program of CAAS, and CARS-12.

## Conflict of Interest

The authors declare that the research was conducted in the absence of any commercial or financial relationships that could be construed as a potential conflict of interesting.

## Publisher’s Note

All claims expressed in this article are solely those of the authors and do not necessarily represent those of their affiliated organizations, or those of the publisher, the editors and the reviewers. Any product that may be evaluated in this article, or claim that may be made by its manufacturer, is not guaranteed or endorsed by the publisher.

## Footnotes

^1^ https://www.genoscope.cns.fr/brassicanapus/

^2^ http://www.geneontology.org

^3^ https://mapman.gabipd.org/mapman/
