# Supplementary material for: Inositol Improves Cold Tolerance Through Inhibiting CBL1 and Increasing Ca2+ Influx in Rapeseed (Brassica napus L.)
Source: Front Plant Sci. 2022 Mar 17;13:775692. doi: 10.3389/fpls.2022.775692 (PMC8969906; doi:10.3389/fpls.2022.775692)
Supplement: Supplementary file 1 [file Data_Sheet_1.docx]

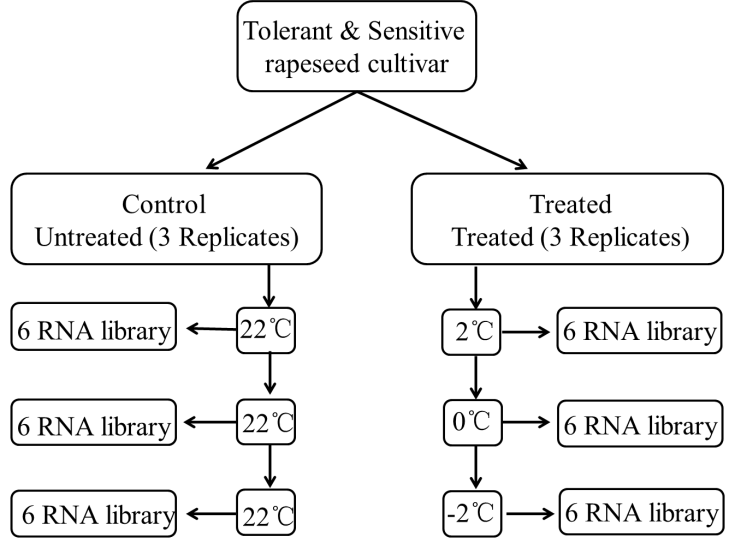


# Fig.S1 The treatment of cold stress on the cold tolerant (C18) and sensitive (C20) rapeseed cultivars for RNA library construction.


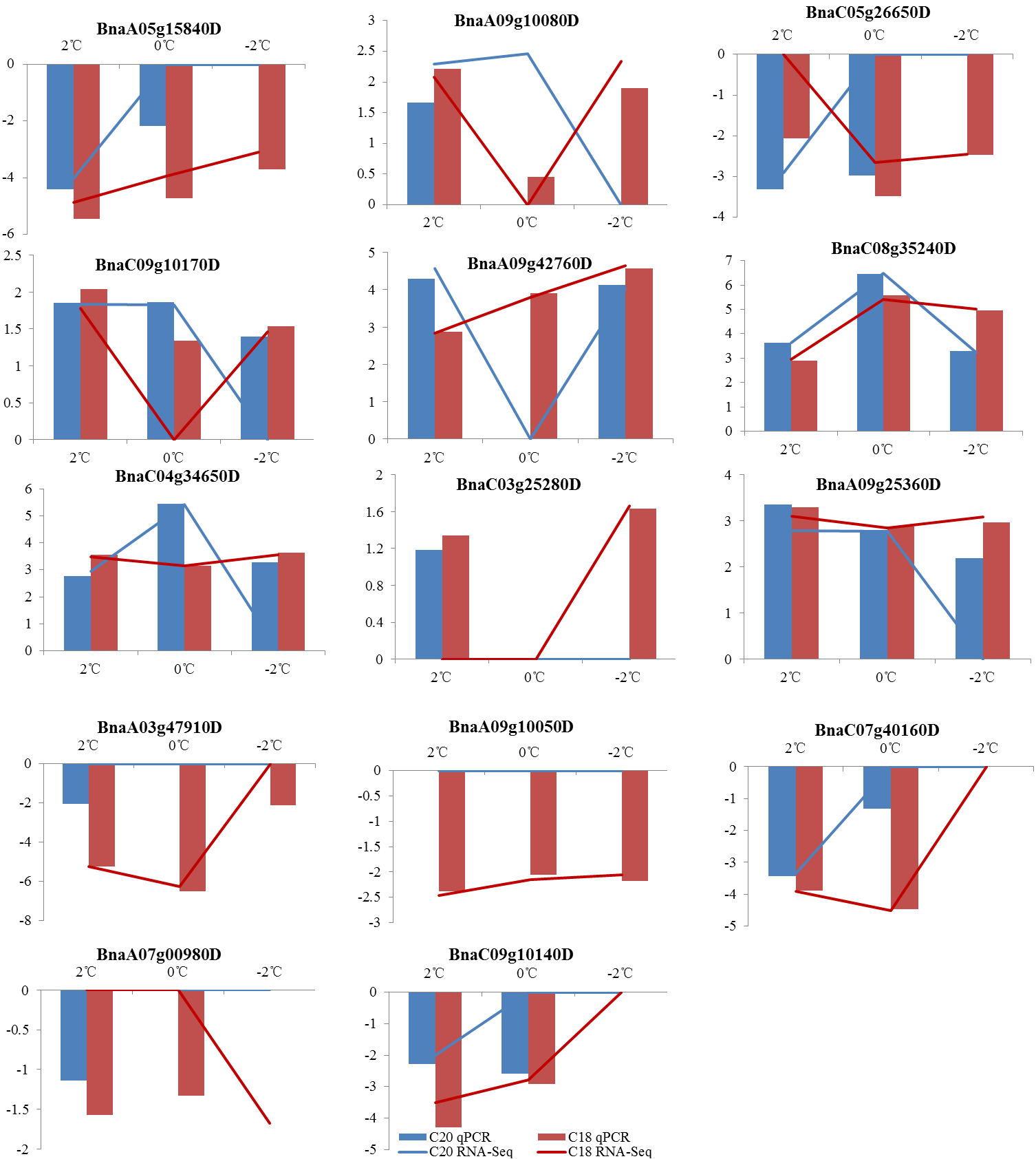


# Fig.S2 The expression pattern of DEGs in inositol pathway by qRT-PCR


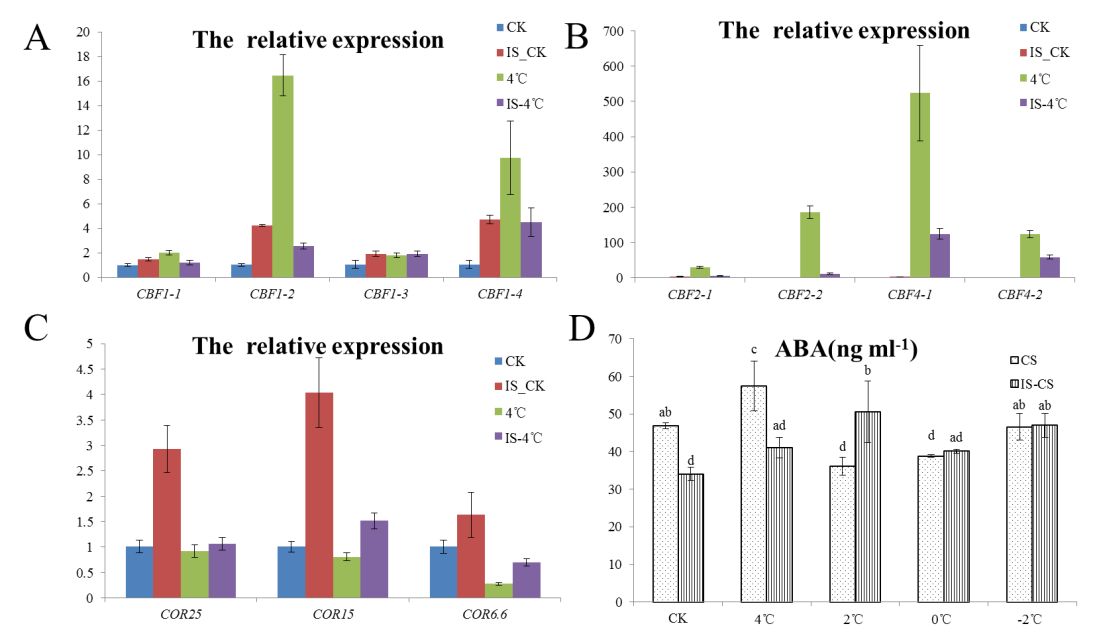


# Fig.S3 Expression patterns of *CBFs*, *CORs* and ABA content with exogenous inositol under cold stress.

A: The expression patterns of *CBF1* (*CBF1-1- CBF1-4*) with exogenous inositol under cold stress; B: The expression patterns of *CBF2* (*CBF2-1* and *CBF2-2*) and *CBF4* (*CBF4-1* and *CBF4-2*) with exogenous inositol under cold stress; C: The expression patterns of *CORs* (*COR25*, *COR15* and *COR6.6*) with exogenous inositol under cold stress; D: ABA content change with exogenous inositol under cold stress. CK: 22°C; 4°C: 4°C treatment for 1h ; IS_CK: 22°C for 1 h with 0.5 g L-1 of exogenous inositol; IS_4°C: 4°C for 1 h with 0.5 g L-1 of exogenous inositol.


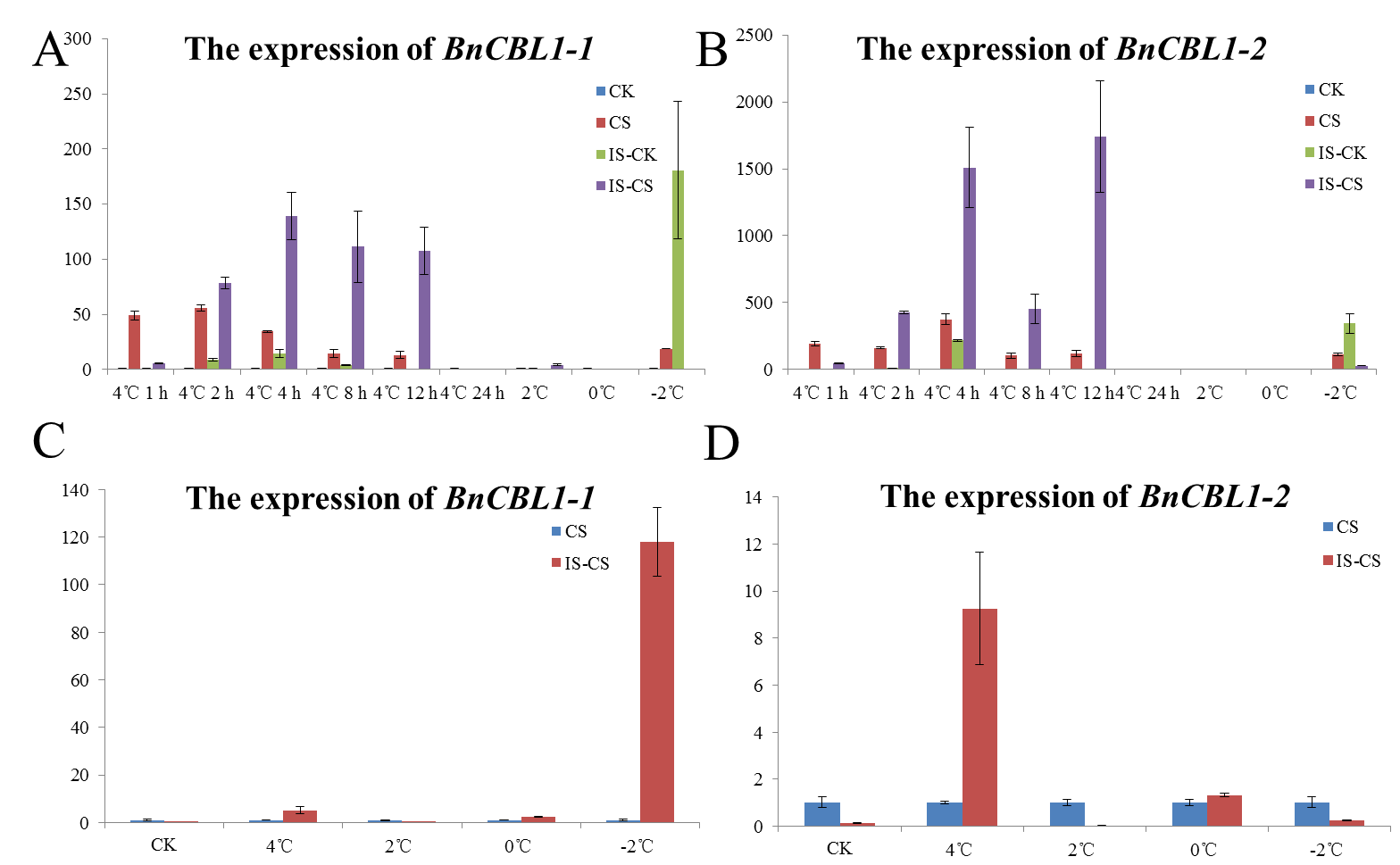


# Fig.S4 The expression patterns of *CBL1s* under cold stress with different pretreatment of exogenous inositol.

A, B: the expression pattern of *CBL1-1* and *CBL1-2* right after the pretreatment of exogenous inositol.

C, D: the expression pattern of *CBL1-1* and *CBL1-2* two days after the pretreatment of exogenous inositol.

| Table S1 The statistics of RNA-Seq for thirty-six B.napus libraries referring to B.napus genome. | | | | | | |
| --- | --- | --- | --- | --- | --- | --- |
| Sample | Q30% | GC content% | Mapped reads | Unique Mapped reads | Raw Data | Valid Data |
| TC2_1 | 97.32 | 49 | 25523786 | 20164591 (79.00%) | 52305176 | 51047572 |
| TC2_2 | 97.48 | 52 | 23362674 | 8914456 (38.16%) | 47802738 | 46725348 |
| TC2_3 | 97.47 | 49 | 21975915 | 16870938 (76.77%) | 46295134 | 43951830 |
| TC0_1 | 97.06 | 49 | 19913235 | 15545728 (78.07%) | 40718782 | 39826470 |
| TC0_2 | 97.46 | 49 | 30537438 | 24058802 (78.78%) | 61864520 | 61074876 |
| TC0_3 | 96.84 | 49 | 20075203 | 15691527 (78.16%) | 40986286 | 40150406 |
| TF2_1 | 95.65 | 49.5 | 19488259 | 14318527 (73.47%) | 40587378 | 38976518 |
| TF2_2 | 96.48 | 49 | 25483578 | 19362316 (75.98%) | 52075526 | 50967156 |
| TF2_3 | 95.19 | 49.5 | 20027720 | 14117183 (70.49%) | 41859862 | 40055440 |
| SC2_1 | 96.08 | 50 | 19850565 | 14680491 (73.96%) | 41166684 | 39701130 |
| SC2_2 | 96.35 | 49 | 20675206 | 16242046 (78.56%) | 42047208 | 41350412 |
| SC2_3 | 95.12 | 50.5 | 22602769 | 14494186 (64.13%) | 47087552 | 45205538 |
| SC0_1 | 95.65 | 51 | 19431750 | 10587590 (54.49%) | 41033690 | 38863500 |
| SC0_2 | 96.48 | 49 | 21470696 | 16584169 (77.24%) | 44296466 | 42941392 |
| SC0_3 | 94.35 | 48.5 | 24553732 | 18898103 (76.97%) | 50738332 | 49107464 |
| SF2_1 | 93.8 | 48.5 | 19031795 | 14535991 (76.38%) | 39919094 | 38063590 |
| SF2_2 | 95.72 | 47.5 | 20152409 | 15676290 (77.79%) | 40991994 | 40304818 |
| SF2_3 | 95.93 | 48 | 20280493 | 15940542 (78.60%) | 41009358 | 40560986 |
| TC2_1_CK | 96.09 | 47 | 20202681 | 15878000 (78.59%) | 40954716 | 40405362 |
| TC2_2_CK | 95.37 | 48.5 | 25605832 | 19328846 (75.49%) | 53892482 | 51211664 |
| TC2_3_CK | 95.48 | 49 | 27457466 | 20552059 (74.85%) | 57992004 | 54914932 |
| TC0_1_CK | 94.09 | 51 | 21880932 | 10690565 (48.86%) | 45268044 | 43761864 |
| TC0_2_CK | 93.26 | 48.5 | 18459015 | 13235630 (71.70%) | 40044530 | 36918030 |
| TC0_3_CK | 93.77 | 48.5 | 18431634 | 13283064 (72.07%) | 41811088 | 36863268 |
| TF2_1_CK | 94.44 | 48.5 | 25812753 | 18942676 (73.38%) | 54634662 | 51625506 |
| TF2_2_CK | 95.02 | 48.5 | 21677876 | 16439076 (75.83%) | 47249848 | 43355752 |
| TF2_3_CK | 95.37 | 49 | 20752353 | 14518590 (69.96%) | 45769662 | 41504706 |
| SC2_1_CK | 94.14 | 48.5 | 18777657 | 13532569 (72.07%) | 41575902 | 37555314 |
| SC2_2_CK | 93.74 | 49 | 21669815 | 15139577 (69.86%) | 49276018 | 43339630 |
| SC2_3_CK | 94.85 | 54 | 26572674 | 4450682 (16.75%) | 54562662 | 53145348 |
| SC0_1_CK | 95.12 | 49 | 24137180 | 17228761 (71.38%) | 50924016 | 48274360 |
| SC0_2_CK | 94.97 | 49.5 | 20790710 | 14198896 (68.29%) | 49793960 | 41581420 |
| SC0_3_CK | 94.43 | 49.5 | 25630366 | 16463084 (64.23%) | 55215144 | 51260732 |
| SF2_1_CK | 97.53 | 50 | 25081551 | 16744924 (66.76%) | 50756270 | 50163102 |
| SF2_2_CK | 95.56 | 47.5 | 22597429 | 17256331 (76.36%) | 46073702 | 45194858 |
| SF2_3_CK | 95.84 | 47 | 26284924 | 20408975 (77.65%) | 53275590 | 52569848 |

# Table S2 DEGs of related CBF and ABA in RNA-seq

T1: the tolerant cultivar C18 under 2°C compared to 22°C; T2: the tolerant cultivar C18 under 0°C compared to 22°C; T3: the tolerant cultivar C18 under -2°C compared to 22°C; S1: the sensitive cultivar C20 under 2°C compared to 22°C; S2: the sensitive cultivar C20 under 0°C compared to 22°C; S3: the sensitive cultivar C20 under -2°C compared to 22°C.

| Gene | Log2(S1) | Log2(S2) | Log2(S3) | Log2(T1) | Log2(T2) | Log2(T3) | Symbol |
| --- | --- | --- | --- | --- | --- | --- | --- |
| BnaA03g13620D | 10.20 | 3.74 | 6.49 | 5.55 | 6.15 | 9.85 | CBF1 |
| BnaAnng34260D | 5.03 | 4.93 | 9.54 | 6.02 | 6.77 | 9.09 | CBF1 |
| BnaA10g07630D | 4.46 | 0.00 | 11.03 | 6.24 | 6.81 | 6.35 | CBF4 |
| BnaA07g12170D | 3.30 | 2.74 | 2.99 | 3.76 | 3.46 | 3.08 | ABA1 |
| BnaC07g16350D | 2.28 | 2.08 | 2.09 | 2.26 | 2.38 | 2.06 | ABA1 |
| BnaC03g69290D | 1.25 | 0.00 | 0.00 | 0.00 | 0.00 | 0.00 | ABA2 |
| BnaC06g41140D | -1.29 | -1.25 | -1.45 | -1.96 | -1.83 | -1.71 | ABA2 |
| BnaA06g38330D | 0.00 | 0.00 | 0.00 | -1.35 | 0.00 | 0.00 | ABA3 |
| BnaC05g12650D | 0.00 | 0.00 | 0.00 | 0.00 | -1.19 | 0.00 | ABA3 |
| BnaC02g17160D | 0.00 | 0.00 | 0.00 | 0.00 | 1.51 | 0.00 | ABA4 |
| BnaA05g08020D | -3.89 | -2.65 | -2.29 | -3.72 | -3.45 | -3.27 | ABI5 |
| BnaA03g35190D | -1.26 | 0.00 | 0.00 | -2.06 | 0.00 | -2.41 | ABI5 |
| BnaA04g23690D | -1.57 | 0.00 | 0.00 | -2.07 | -1.51 | -1.05 | ABI5 |
| BnaA06g03040D | 0.00 | 2.93 | 1.90 | 0.00 | 1.12 | 0.00 | ABI5 |
| BnaA09g36130D | -1.97 | 0.00 | -1.15 | 0.00 | 0.00 | -1.16 | ABI5 |
| BnaA10g28780D | 0.00 | 1.29 | 0.00 | 0.00 | 1.59 | 1.09 | ABI5 |
| BnaAnng26550D | 0.00 | 0.00 | -1.19 | 0.00 | -1.30 | 0.00 | ABI5 |
| BnaC04g52290D | -3.76 | 0.00 | 0.00 | -5.57 | 0.00 | 0.00 | ABI5 |
| BnaC04g56840D | -2.65 | -1.82 | -2.91 | -5.87 | -2.61 | -3.31 | ABI5 |
| BnaC06g02650D | 1.91 | 2.44 | 1.96 | 2.05 | 2.14 | 2.78 | ABI5 |
| BnaC07g44670D | 0.00 | 2.08 | 0.00 | 0.00 | 0.00 | 0.00 | ABI5 |
| BnaC08g27660D | -2.37 | -1.33 | -1.87 | -1.93 | -1.32 | -1.44 | ABI5 |
| BnaCnng41320D | -1.55 | -2.18 | -2.19 | -2.01 | -1.83 | -2.13 | ABI5 |

| Table S3 Ca2+-related genes in the RNAseq data | | | | | | | | |
| --- | --- | --- | --- | --- | --- | --- | --- | --- |
| Gene ID | Gene ID in Arabidopsis | Log2(S1) | Log2(S2) | Log2(S3) | Log2(T1) | Log2(T2) | Log2(T3) | description |
| BnaA09g48890D | at1g08450 | #N/A | 2.7889655 | #N/A | -2.793948 | -3.261159 | -3.051655 | Encodes one of three Arabidopsis calreticulins |
| BnaC08g17850D | at1g18210 | #N/A | -1.320885 | #N/A | 1.2520334 | #N/A | #N/A | Calcium-binding EF-hand family protein |
| BnaA06g38470D | at1g18210 | #N/A | -1.032423 | 1.4784575 | 1.7754823 | -1.157997 | #N/A | Calcium-binding EF-hand family protein |
| BnaA08g22720D | at1g18210 | #N/A | -2.37975 | #N/A | 1.5017108 | #N/A | #N/A | Calcium-binding EF-hand family protein |
| BnaA09g28330D | at1g24620 | #N/A | #N/A | -2.350752 | -2.254592 | 1.5153793 | #N/A | EF hand calcium-binding protein family |
| BnaAnng04600D | at1g35670 | #N/A | 1.8029788 | #N/A | -1.432803 | -1.370618 | #N/A | Encodes a Ca2+ |
| BnaA07g39090D | at1g73805 | 4.053674 | 6.227499 | #N/A | -3.90648 | -5.058417 | -5.924421 | Calmodulin binding protein-like |
| BnaC06g34630D | at1g73805 | #N/A | 4.168332 | 1.9278114 | -3.493359 | -4.277477 | -4.328908 | Calmodulin binding protein-like |
| BnaA07g22510D | at1g73805 | 3.6644766 | 5.479371 | #N/A | -2.896337 | -2.401301 | -4.121769 | Calmodulin binding protein-like |
| BnaC06g23400D | at1g73805 | 2.7396252 | #N/A | #N/A | -2.358522 | -2.627612 | -4.985979 | Calmodulin binding protein-like |
| BnaC07g21900D | at2g02790 | #N/A | 6.445315 | #N/A | #N/A | #N/A | -1.518765 | IQ-domain 29 IQD29 |
| BnaC02g34780D | at2g02790 | #N/A | #N/A | #N/A | #N/A | 1.4309636 | -1.625196 | IQ-domain 29 IQD29 |
| BnaA04g19410D | at2g33380 | -1.083275 | #N/A | #N/A | #N/A | #N/A | 2.383042 | Encodes a calcium binding protein |
| BnaA05g06290D | at2g38800 | #N/A | 3.8887358 | #N/A | -1.828148 | -1.409321 | -1.756489 | Plant calmodulin-binding protein-related |
| BnaC04g56800D | at2g41010 | #N/A | -2.43062 | #N/A | 3.050586 | 2.5315688 | 2.5595648 | Encodes a novel calmodulin binding protein |
| BnaC07g25470D | at3g29000 | #N/A | 2.6216145 | #N/A | -2.452291 | -4.158116 | -5.492331 | Calcium-binding EF-hand family protein |
| BnaA06g17340D | at3g47480 | #N/A | 4.2151914 | #N/A | #N/A | -4.085476 | -5.088983 | Calcium-binding EF-hand family protein |
| BnaA03g40940D | at3g50770 | #N/A | 2.1600308 | #N/A | -3.283339 | -2.712702 | -3.181491 | calmodulin-like 41 CML41 |
| BnaA03g19320D | at3g56800 | #N/A | #N/A | 1.519364 | 1.7001158 | -1.024624 | #N/A | encodes a calmodulin |
| BnaA09g36820D | at3g57530 | 1.4420943 | #N/A | 2.9886477 | #N/A | #N/A | -2.950234 | Calcium-dependent Protein Kinase |
| BnaA01g17480D | at4g16350 | #N/A | #N/A | #N/A | #N/A | #N/A | -1.552218 | Calcium sensor protein. Binds CIPK14. |
| BnaC01g21810D | at4g16350 | #N/A | #N/A | #N/A | -2.765864 | #N/A | #N/A | Calcium sensor protein. Binds CIPK14. |
| BnaC07g34450D | at4g17615 | #N/A | #N/A | 1.9201429 | #N/A | -2.144587 | #N/A | Member of AtCBL Calcineurin B-like Calcium Sensor Proteins |
| BnaA03g43160D | at4g17615 | #N/A | #N/A | 1.7391135 | #N/A | #N/A | #N/A | Member of AtCBL Calcineurin B-like Calcium Sensor Proteins |
| BnaA01g08510D | at4g17615 | #N/A | #N/A | 4.230346 | #N/A | #N/A | -2.603583 | Member of AtCBL Calcineurin B-like Calcium Sensor Proteins |
| BnaC01g10210D | at4g17615 | 1.5672282 | #N/A | 4.111653 | #N/A | #N/A | #N/A | Member of AtCBL Calcineurin B-like Calcium Sensor Proteins |
| BnaA01g12870D | at4g23060 | #N/A | -1.137717 | #N/A | 2.066089 | #N/A | 2.6555696 | IQ-domain 22 IQD22 |
| BnaA03g52310D | at4g33000 | -3.799926 | #N/A | -3.793051 | #N/A | #N/A | #N/A | Encodes a member of the calcineurin B-like calcium sensor gene family |
| BnaC07g44060D | at4g33000 | #N/A | -1.245873 | #N/A | -1.914124 | #N/A | -1.10483 | Encodes a member of the calcineurin B-like calcium sensor gene family |
| BnaC03g66810D | at4g33000 | #N/A | -1.46003 | #N/A | #N/A | #N/A | #N/A | Encodes a member of the calcineurin B-like calcium sensor gene family |
| BnaC01g05180D | at4g33050 | 4.6026964 | #N/A | 3.4630613 | #N/A | #N/A | -3.74599 | embryo sac development arrest 39 EDA39 |
| BnaA01g02980D | at4g34150 | #N/A | #N/A | 1.0311 | #N/A | -1.487185 | #N/A | Calcium-dependent lipid-binding CaLB domain |
| BnaAnng00580D | at5g03040 | #N/A | -1.660407 | #N/A | 1.0542135 | #N/A | #N/A | IQ-domain 2 iqd2 |
| BnaC02g03410D | at5g03040 | -1.317193 | #N/A | 2.567955 | #N/A | #N/A | #N/A | IQ-domain 2 iqd2 |
| BnaA03g01490D | at5g04870 | 3.6444929 | 1.781949 | 1.837427 | #N/A | 1.3123952 | -1.418655 | A calcium-dependent protein kinase that can phosphorylate phenylalanine ammonia lyase PAL |
| BnaC09g43910D | at5g26920 | #N/A | -1.435249 | -3.086263 | #N/A | #N/A | 5.2826023 | Encodes a calmodulin-binding protein CBP60g calmodulin binding protein 60-like.g |
| BnaA02g31430D | at5g26920 | #N/A | 4.469833 | #N/A | -3.129868 | -4.215536 | -2.275427 | Encodes a calmodulin-binding protein CBP60g calmodulin binding protein 60-like.g |
| BnaC02g40040D | at5g26920 | 1.3475189 | 3.2147126 | 4.1322913 | -1.776213 | -3.281656 | -2.031177 | Encodes a calmodulin-binding protein CBP60g calmodulin binding protein 60-like.g |
| BnaA09g03490D | at5g28830 | -1.032748 | #N/A | #N/A | #N/A | #N/A | 1.1664072 | calcium-binding EF hand family protein |
| BnaC06g12520D | at5g39670 | #N/A | 3.4150476 | #N/A | -3.06141 | -2.057497 | -2.812939 | Calcium-binding EF-hand family protein |
| BnaC09g16820D | at5g42380 | #N/A | 2.8809915 | 3.5050097 | #N/A | -2.839063 | -3.738837 | calmodulin like 37 CML37 |
| BnaAnng31750D | at5g47100 | #N/A | #N/A | 1.2600976 | #N/A | #N/A | #N/A | member of AtCBLs Calcineurin B-like Calcium Sensor Proteins |
| BnaA02g24990D | at5g47100 | 1.9302002 | 1.5270222 | 1.7835203 | 1.1776984 | 1.0420381 | 2.1175146 | member of AtCBLs Calcineurin B-like Calcium Sensor Proteins |
| BnaC03g13890D | at5g55990 | #N/A | #N/A | #N/A | #N/A | 1.6440068 | 3.1035893 | Encodes a member of the Arabidopsis CBL Calcineurin B-like Calcium Sensor |
| BnaC02g12710D | at5g55990 | -1.198631 | #N/A | #N/A | -1.791308 | -1.422892 | -1.163261 | Encodes a member of the Arabidopsis CBL Calcineurin B-like Calcium Sensor |
| BnaA03g10900D | at5g56360 | #N/A | 4.326409 | #N/A | -1.811106 | -1.322354 | #N/A | Encodes PSL4, beat-subunit of endoplasmic reticulum-resident glucosidase II |
| BnaC03g52210D | at5g61910 | 1.4266603 | 3.9132142 | 3.3535798 | -1.771666 | -1.936039 | -2.25542 | DCD Development and Cell Death domain protein |
| BnaA06g21360D | at5g61910 | 2.1110122 | 3.6931145 | #N/A | -2.479957 | -2.938088 | -4.196309 | DCD Development and Cell Death domain protein |
| BnaC03g45210D | at5g37780 | 1.5286338 | 4.091193 | #N/A | -2.087019 | -2.525183 | -2.420098 | Calmodulin-related protein |
| BnaC05g00790D | at1g01110 | -1.00752 | #N/A | #N/A | #N/A | #N/A | #N/A | IQ-domain 18 IQD18 |
| BnaA10g00710D | at1g01110 | #N/A | #N/A | -1.184648 | #N/A | #N/A | #N/A | IQ-domain 18 IQD18 |
| BnaA09g51440D | at1g02270 | 2.5503156 | #N/A | 4.028498 | #N/A | #N/A | #N/A | Calcium-binding endonuclease/exonuclease/phosphatase family |
| BnaA10g00930D | at1g02270 | #N/A | 2.517266 | 1.563644 | 1.5353616 | 1.2984815 | 1.3148056 | Calcium-binding endonuclease/exonuclease/phosphatase family |
| BnaC05g03160D | at1g05150 | -1.05037 | -1.388396 | -1.184126 | #N/A | #N/A | -1.014054 | Calcium-binding tetratricopeptide family protein |
| BnaA10g03170D | at1g05150 | #N/A | -1.335969 | #N/A | #N/A | #N/A | #N/A | Calcium-binding tetratricopeptide family protein |
| BnaC08g13550D | at1g08450 | #N/A | #N/A | #N/A | -2.059618 | -2.282136 | -2.090053 | Encodes one of three Arabidopsis calreticulins |
| BnaA08g26940D | at1g08450 | #N/A | #N/A | -1.363315 | -1.354964 | -1.993007 | -2.355279 | Encodes one of three Arabidopsis calreticulins |
| BnaC08g43170D | at1g08450 | #N/A | #N/A | #N/A | -2.375866 | -2.215294 | -2.971461 | Encodes one of three Arabidopsis calreticulins |
| BnaC08g42940D | at1g08860 | #N/A | #N/A | #N/A | #N/A | #N/A | -5.23438 | Encodes a copine-like protein |
| BnaA09g48680D | at1g08860 | -4.970567 | #N/A | #N/A | -3.094065 | -5.724321 | -3.969463 | Encodes a copine-like protein |
| BnaCnng06700D | at1g09210 | 3.087646 | 3.8517487 | #N/A | #N/A | 2.1522193 | #N/A | Encodes one of three Arabidopsis calreticulins. |
| BnaA06g05290D | at1g09210 | 1.8508383 | 1.4936174 | 1.945095 | 1.480081 | #N/A | 1.2648461 | Encodes one of three Arabidopsis calreticulins. |
| BnaC05g06780D | at1g09210 | 1.780444 | 2.4545617 | 2.3661835 | 1.9111718 | 1.2060742 | 1.7845814 | Encodes one of three Arabidopsis calreticulins. |
| BnaA08g26530D | at1g09210 | 3.6658158 | 2.566211 | 2.201497 | #N/A | 1.4156083 | 3.0653906 | Encodes one of three Arabidopsis calreticulins. |
| BnaC07g00750D | at1g09210 | 2.2075071 | #N/A | #N/A | #N/A | #N/A | #N/A | Encodes one of three Arabidopsis calreticulins. |
| BnaC08g15540D | at1g12310 | #N/A | -1.274975 | #N/A | #N/A | #N/A | #N/A | Calcium-binding EF-hand family protein |
| BnaA06g09210D | at1g14380 | -1.932994 | -2.12207 | -1.093474 | -2.422837 | #N/A | -1.823058 | IQ-domain 28 IQD28 |
| BnaC05g10620D | at1g14380 | -2.306676 | -1.866219 | -1.790336 | -2.440627 | #N/A | -2.122793 | IQ-domain 28 IQD28 |
| BnaC05g13970D | at1g18210 | #N/A | -2.242907 | #N/A | #N/A | -1.968162 | -1.119534 | Calcium-binding EF-hand family protein |
| BnaA08g22360D | at1g18840 | #N/A | #N/A | #N/A | -2.012442 | #N/A | -1.325678 | IQ-domain 30 IQD30 |
| BnaC05g14520D | at1g18840 | #N/A | 2.1018708 | #N/A | #N/A | 1.4502255 | #N/A | IQ-domain 30 IQD30 |
| BnaA09g44420D | at1g18890 | #N/A | #N/A | #N/A | #N/A | -1.150777 | #N/A | encodes a calcium-dependent protein kinase |
| BnaC05g14600D | at1g18890 | -2.744942 | -1.35237 | #N/A | #N/A | -2.488403 | -1.030952 | encodes a calcium-dependent protein kinase |
| BnaC08g36980D | at1g18890 | -1.545793 | #N/A | #N/A | #N/A | -1.183133 | #N/A | encodes a calcium-dependent protein kinase |
| BnaC05g15430D | at1g19870 | -2.46491 | -2.407028 | -1.863739 | -2.516984 | #N/A | -2.366646 | IQ-domain 32 iqd32 |
| BnaA06g14070D | at1g19870 | -1.764648 | -2.177135 | #N/A | -2.073558 | -1.269035 | -1.953933 | IQ-domain 32 iqd32 |
| BnaC08g19060D | at1g19870 | -1.339421 | #N/A | -1.281987 | -1.628786 | #N/A | -1.062205 | IQ-domain 32 iqd32 |
| BnaA08g31270D | at1g19870 | -1.176375 | -1.249832 | #N/A | -1.014946 | #N/A | #N/A | IQ-domain 32 iqd32 |
| BnaA08g21680D | at1g20760 | 1.0961462 | #N/A | #N/A | #N/A | #N/A | 1.360509 | Calcium-binding EF hand family protein |
| BnaC08g19440D | at1g20760 | #N/A | #N/A | 1.5849136 | #N/A | #N/A | #N/A | Calcium-binding EF hand family protein |
| BnaA06g15280D | at1g21550 | #N/A | #N/A | #N/A | -4.965162 | -5.643448 | -6.101682 | Calcium-binding EF-hand family protein |
| BnaC05g20880D | at1g24620 | -3.508331 | #N/A | #N/A | #N/A | #N/A | #N/A | EF hand calcium-binding protein family |
| BnaC03g57710D | at1g27460 | #N/A | -2.152517 | -2.127242 | #N/A | #N/A | #N/A | encodes a calmodulin-binding protein |
| BnaA09g27080D | at1g29025 | #N/A | #N/A | #N/A | #N/A | -1.000218 | #N/A | Calcium-binding EF-hand family protein |
| BnaC06g09680D | at1g52410 | 2.0993018 | 6.888326 | 2.0396357 | #N/A | #N/A | #N/A | Contains a novel calcium-binding repeat sequence |
| BnaA05g14880D | at1g52410 | #N/A | 4.5855393 | #N/A | #N/A | #N/A | #N/A | Contains a novel calcium-binding repeat sequence |
| BnaA05g14600D | at1g53210 | -2.251437 | -2.55864 | -1.217983 | -1.263344 | -3.495117 | -1.633659 | sodium/calcium exchanger family protein / calcium-binding EF hand family protein |
| BnaCnng29300D | at1g53210 | -2.571731 | -3.151624 | -1.407389 | #N/A | -3.644798 | -1.417993 | sodium/calcium exchanger family protein / calcium-binding EF hand family protein |
| BnaA06g01060D | at1g53210 | -1.587041 | -2.469002 | -1.712079 | #N/A | -2.478477 | -1.147966 | sodium/calcium exchanger family protein / calcium-binding EF hand family protein |
| BnaC03g69500D | at1g53210 | -1.313501 | -2.789213 | -2.022413 | #N/A | #N/A | -1.582698 | sodium/calcium exchanger family protein / calcium-binding EF hand family protein |
| BnaA08g01270D | at1g53210 | -1.911209 | -2.589715 | -2.537957 | -1.287801 | -1.450902 | -1.617362 | sodium/calcium exchanger family protein / calcium-binding EF hand family protein |
| BnaC09g16670D | at1g54530 | #N/A | #N/A | 1.4619256 | #N/A | #N/A | #N/A | Calcium-binding EF hand family protein |
| BnaC01g43040D | at1g56340 | 1.2959293 | 1.3102225 | 2.0302906 | #N/A | #N/A | #N/A | Encodes one of three Arabidopsis calreticulins |
| BnaA09g15970D | at1g56340 | #N/A | #N/A | 1.6063464 | #N/A | #N/A | #N/A | Encodes one of three Arabidopsis calreticulins |
| BnaA09g15400D | at1g56340 | 1.9048734 | 1.9436169 | 1.9265624 | 1.3023719 | #N/A | #N/A | Encodes one of three Arabidopsis calreticulins |
| BnaC09g16150D | at1g56340 | 1.347056 | #N/A | 1.5387114 | 1.0501782 | #N/A | #N/A | Encodes one of three Arabidopsis calreticulins |
| BnaAnng19720D | at1g64480 | #N/A | #N/A | #N/A | #N/A | #N/A | -2.260795 | calcineurin B-like protein 8 |
| BnaA02g14260D | at1g64850 | #N/A | 1.3228697 | 1.0602169 | 1.2292717 | #N/A | 1.016924 | Calcium-binding EF hand family protein |
| BnaA07g30380D | at1g72670 | #N/A | #N/A | #N/A | #N/A | 6.315123 | #N/A | IQ-domain 8 iqd8 |
| BnaAnng32190D | at1g73440 | #N/A | #N/A | #N/A | -1.132513 | #N/A | #N/A | calmodulin-related |
| BnaA07g30990D | at1g73630 | 3.7423105 | 3.2346025 | #N/A | #N/A | #N/A | #N/A | EF hand calcium-binding protein family |
| BnaC06g34440D | at1g73630 | 3.1292734 | 2.0385349 | #N/A | 2.2437518 | 1.5613043 | 2.9427524 | EF hand calcium-binding protein family |
| BnaC06g36670D | at1g76040 | #N/A | #N/A | #N/A | #N/A | 2.1950042 | #N/A | member of Calcium Dependent Protein Kinase |
| BnaCnng52460D | at1g76040 | #N/A | #N/A | 2.0913699 | #N/A | #N/A | #N/A | member of Calcium Dependent Protein Kinase |
| BnaC06g37490D | at1g76650 | 3.9960372 | #N/A | 7.214596 | 4.175993 | 3.3909254 | 3.8699632 | calmodulin-like 38 CML38 |
| BnaCnng76780D | at1g76650 | #N/A | #N/A | 5.686307 | 5.643509 | #N/A | 3.32996 | calmodulin-like 38 CML38 |
| BnaC09g21100D | at2g02790 | -1.052328 | #N/A | #N/A | #N/A | #N/A | #N/A | IQ-domain 29 IQD29 |
| BnaA02g36860D | at2g02790 | #N/A | #N/A | #N/A | #N/A | 1.0123274 | #N/A | IQ-domain 29 IQD29 |
| BnaCnng52620D | at2g15680 | -1.256209 | #N/A | #N/A | -2.271598 | -2.625265 | -2.43501 | Calcium-binding EF-hand family protein |
| BnaC03g47560D | at2g17290 | #N/A | #N/A | 1.734502 | #N/A | #N/A | #N/A | Encodes calcium dependent protein kinase 6 CPK6 |
| BnaA06g25880D | at2g17290 | #N/A | 2.327594 | 2.5054832 | #N/A | #N/A | #N/A | Encodes calcium dependent protein kinase 6 CPK6 |
| BnaC09g09270D | at2g17290 | #N/A | #N/A | #N/A | #N/A | -1.58009 | #N/A | Encodes calcium dependent protein kinase 6 CPK6 |
| BnaCnng22820D | at2g17290 | -1.595629 | #N/A | #N/A | #N/A | #N/A | #N/A | Encodes calcium dependent protein kinase 6 CPK6 |
| BnaA09g09050D | at2g17290 | -1.441605 | #N/A | #N/A | #N/A | -1.362688 | #N/A | Encodes calcium dependent protein kinase 6 CPK6 |
| BnaA09g09750D | at2g18750 | 1.8018538 | 1.9628102 | 1.1211503 | 1.6675937 | 1.3507336 | 1.8660369 | Calmodulin-binding protein |
| BnaCnng22620D | at2g18750 | #N/A | 1.1998837 | 1.3824853 | 1.0376031 | 1.1172282 | 1.5119189 | Calmodulin-binding protein |
| BnaA04g15340D | at2g26180 | #N/A | #N/A | 2.9481356 | #N/A | #N/A | #N/A | IQ-domain 6 IQD6 |
| BnaC04g38300D | at2g26190 | #N/A | #N/A | #N/A | -1.003529 | -1.257227 | -2.634926 | calmodulin-binding family protein |
| BnaA04g15350D | at2g26190 | #N/A | #N/A | #N/A | #N/A | #N/A | -1.643123 | calmodulin-binding family protein |
| BnaA09g40510D | at2g26410 | #N/A | #N/A | #N/A | -3.404077 | #N/A | #N/A | IQ-domain 4 Iqd4 |
| BnaC04g38550D | at2g27030 | #N/A | #N/A | #N/A | 1.0357914 | #N/A | #N/A | encodes a calmodulin |
| BnaA04g15550D | at2g27030 | 1.4080122 | 2.8188202 | 2.4860373 | 3.1191099 | 1.8909986 | 2.3736784 | encodes a calmodulin |
| BnaC03g47120D | at2g27480 | #N/A | #N/A | #N/A | -1.10019 | #N/A | #N/A | Calcium-binding EF-hand family protein |
| BnaA03g22380D | at2g27480 | -1.609812 | #N/A | -1.110068 | #N/A | #N/A | #N/A | Calcium-binding EF-hand family protein |
| BnaA03g15390D | at2g33380 | 1.7076017 | #N/A | #N/A | #N/A | #N/A | 4.947732 | Encodes a calcium binding protein |
| BnaC03g18600D | at2g33380 | #N/A | 5.713253 | #N/A | #N/A | 2.6287065 | #N/A | Encodes a calcium binding protein |
| BnaCnng05100D | at2g33990 | #N/A | 1.0107267 | #N/A | #N/A | #N/A | #N/A | IQ-domain 9 iqd9 |
| BnaA05g09740D | at2g33990 | 1.1591674 | 2.033563 | 1.8918136 | #N/A | 1.2754223 | #N/A | IQ-domain 9 iqd9 |
| BnaC04g06540D | at2g38800 | #N/A | #N/A | #N/A | #N/A | #N/A | -1.331724 | Plant calmodulin-binding protein-related |
| BnaA03g19330D | at2g41140 | -1.879697 | #N/A | #N/A | #N/A | #N/A | #N/A | Encodes CDPK-related kinase 1 CRK1 |
| BnaC03g23150D | at2g41140 | #N/A | #N/A | #N/A | -1.035778 | #N/A | #N/A | Encodes CDPK-related kinase 1 CRK1 |
| BnaC04g47760D | at2g41560 | #N/A | -1.358891 | #N/A | #N/A | #N/A | -1.409661 | encodes a calmodulin-regulated Ca2+ |
| BnaA04g24000D | at2g41560 | -1.832881 | -2.908993 | -1.487044 | -2.664078 | -1.613029 | -2.896412 | encodes a calmodulin-regulated Ca2+ |
| BnaC03g24000D | at2g43040 | 1.6604377 | #N/A | #N/A | #N/A | #N/A | #N/A | encodes a calmodulin-binding protein |
| BnaA03g20180D | at2g43290 | #N/A | #N/A | 2.1139545 | 1.1659209 | #N/A | #N/A | Encodes calmodulin-like MSS3. |
| BnaA04g25000D | at2g43290 | #N/A | -2.432378 | 1.882443 | #N/A | #N/A | #N/A | Encodes calmodulin-like MSS3. |
| BnaC04g48840D | at2g43290 | -1.564975 | #N/A | #N/A | #N/A | #N/A | #N/A | Encodes calmodulin-like MSS3. |
| BnaC04g49190D | at2g43680 | -3.136319 | -2.425308 | -2.572059 | -3.035612 | -2.027438 | -2.426909 | IQ-domain 14 IQD14 |
| BnaA05g03470D | at2g43680 | -1.323193 | -1.328072 | -1.025807 | -1.420309 | -1.09471 | -1.292712 | IQ-domain 14 IQD14 |
| BnaC04g53080D | at2g43680 | -1.088676 | -1.061815 | #N/A | -2.82307 | #N/A | -2.640935 | IQ-domain 14 IQD14 |
| BnaC03g24390D | at2g43680 | -2.51692 | #N/A | -2.012396 | -2.311181 | -1.062432 | -2.692196 | IQ-domain 14 IQD14 |
| BnaC03g24790D | at2g44310 | -1.05144 | -1.704011 | #N/A | #N/A | #N/A | #N/A | Calcium-binding EF-hand family protein |
| BnaC03g25360D | at2g45670 | #N/A | 1.0620083 | #N/A | 1.1412011 | #N/A | 1.1173389 | calcineurin B subunit-related |
| BnaC04g50380D | at2g45670 | 1.4288814 | 1.0362927 | 1.3817673 | 1.6256168 | 1.1328641 | 1.3403518 | calcineurin B subunit-related |
| BnaA03g51150D | at2g46600 | -2.41185 | -3.83209 | #N/A | #N/A | -2.252738 | #N/A | Calcium-binding EF-hand family protein |
| BnaC03g25880D | at2g46600 | -1.650547 | #N/A | -1.089807 | -2.903326 | -3.075205 | -2.753856 | Calcium-binding EF-hand family protein |
| BnaA04g27150D | at2g46600 | -2.367309 | -1.412756 | #N/A | #N/A | -2.028225 | -1.704172 | Calcium-binding EF-hand family protein |
| BnaC04g51710D | at2g46700 | 1.065284 | #N/A | #N/A | #N/A | #N/A | -2.301586 | CDPK-related kinase 3 CRK3 |
| BnaA03g27250D | at3g01830 | 5.4169083 | #N/A | #N/A | 1.9262439 | #N/A | #N/A | Calcium-binding EF-hand family protein |
| BnaA06g03170D | at3g03410 | -4.395859 | -3.452061 | -2.980571 | -5.081422 | -4.49654 | -3.511633 | EF hand calcium-binding protein family |
| BnaC05g47600D | at3g03950 | 2.12081 | #N/A | #N/A | #N/A | 1.3998761 | 1.1504995 | Physically interacts with CIPK1 |
| BnaC01g40360D | at3g03950 | 1.4121556 | 2.1876047 | #N/A | #N/A | 2.5855265 | #N/A | Physically interacts with CIPK1 |
| BnaA01g33850D | at3g03950 | #N/A | 2.8112848 | 2.4167988 | 2.8945048 | 1.9041877 | 2.2788298 | Physically interacts with CIPK1 |
| BnaA01g32120D | at3g09710 | -1.743132 | -1.546907 | -1.756988 | -1.625104 | -2.767568 | -1.837508 | Ca(2+)-dependent calmodulin-binding protein |
| BnaA01g31850D | at3g10300 | -2.285438 | #N/A | #N/A | #N/A | -1.214155 | #N/A | Calcium-binding EF-hand family protein |
| BnaC01g38680D | at3g10300 | -1.038445 | -1.913941 | #N/A | #N/A | #N/A | #N/A | Calcium-binding EF-hand family protein |
| BnaA03g31330D | at3g10660 | #N/A | #N/A | #N/A | #N/A | #N/A | 1.2316271 | predicted to encode calcium-dependent protein kinase |
| BnaC05g42370D | at3g10660 | #N/A | #N/A | #N/A | #N/A | -1.124641 | #N/A | predicted to encode calcium-dependent protein kinase |
| BnaA05g28200D | at3g10660 | #N/A | #N/A | #N/A | #N/A | 1.8007128 | #N/A | predicted to encode calcium-dependent protein kinase |
| BnaCnng01040D | at3g13460 | 1.9066269 | 1.9511741 | #N/A | 1.3347462 | #N/A | #N/A | Physically interacts with CIPK1. |
| BnaA03g32830D | at3g13460 | 1.1889943 | 1.1756425 | 1.4623666 | 1.6055024 | 1.098163 | 1.3006188 | Physically interacts with CIPK1. |
| BnaC03g37950D | at3g13460 | #N/A | 1.1602191 | 1.4918696 | 1.3796424 | #N/A | 1.0997643 | Physically interacts with CIPK1. |
| BnaC01g36720D | at3g14590 | -1.806681 | #N/A | #N/A | #N/A | -2.074435 | -1.373481 | NTMC2T6.2 |
| BnaA01g29290D | at3g14590 | -2.395677 | -3.221428 | #N/A | -1.608252 | -1.618152 | -3.192801 | NTMC2T6.2 |
| BnaA03g34540D | at3g17470 | #N/A | 2.9233484 | 4.790926 | #N/A | #N/A | #N/A | Ca2+-activated RelA/spot homolog CRSH |
| BnaC03g40030D | at3g17470 | 1.1969988 | #N/A | #N/A | 1.0018301 | #N/A | #N/A | Ca2+-activated RelA/spot homolog CRSH |
| BnaA04g19990D | at3g18430 | -1.077479 | #N/A | #N/A | #N/A | #N/A | #N/A | Calcium-binding EF-hand family protein |
| BnaCnng41460D | at3g18430 | -1.81872 | #N/A | -1.865319 | -3.326506 | -1.51753 | -2.065049 | Calcium-binding EF-hand family protein |
| BnaC04g44380D | at3g18430 | -1.251164 | #N/A | #N/A | #N/A | #N/A | #N/A | Calcium-binding EF-hand family protein |
| BnaA05g18760D | at3g20290 | #N/A | 1.2170793 | #N/A | #N/A | #N/A | #N/A | Encodes AtEHD1, one of the Arabidopsis Eps15 homology domain proteins involved in endocytosis AtEHD2, At4g05520 |
| BnaC01g32680D | at3g20290 | #N/A | #N/A | #N/A | -1.00588 | #N/A | #N/A | Encodes AtEHD1, one of the Arabidopsis Eps15 homology domain proteins involved in endocytosis AtEHD2, At4g05520 |
| BnaC01g32650D | at3g20290 | #N/A | #N/A | -1.345123 | -1.409866 | #N/A | #N/A | Encodes AtEHD1, one of the Arabidopsis Eps15 homology domain proteins involved in endocytosis AtEHD2, At4g05520 |
| BnaA03g36530D | at3g22190 | #N/A | #N/A | #N/A | -1.153962 | #N/A | -1.2634 | IQ-domain 5 IQD5 |
| BnaA01g24830D | at3g22190 | #N/A | #N/A | 2.309999 | #N/A | #N/A | #N/A | IQ-domain 5 IQD5 |
| BnaCnng33700D | at3g22190 | #N/A | #N/A | #N/A | 1.8472903 | 1.5640451 | #N/A | IQ-domain 5 IQD5 |
| BnaC05g30430D | at3g22190 | #N/A | #N/A | #N/A | #N/A | 1.0656964 | #N/A | IQ-domain 5 IQD5 |
| BnaC03g42590D | at3g22190 | 1.5689396 | 3.9727955 | 2.8452148 | 1.7839264 | 1.644484 | 3.0288954 | IQ-domain 5 IQD5 |
| BnaA03g36550D | at3g22190 | #N/A | #N/A | 2.153188 | #N/A | #N/A | #N/A | IQ-domain 5 IQD5 |
| BnaA05g17440D | at3g22190 | 1.1083859 | 1.6906171 | 1.5134588 | 1.3273146 | #N/A | 1.642426 | IQ-domain 5 IQD5 |
| BnaA01g24320D | at3g22930 | -2.492902 | -3.700623 | -3.225515 | -2.17429 | -2.701685 | #N/A | Encodes a calmodulin-like protein. |
| BnaC01g31320D | at3g22930 | -3.297322 | -3.517745 | #N/A | -3.997484 | -3.691265 | -2.684131 | Encodes a calmodulin-like protein. |
| BnaA07g05960D | at3g24110 | #N/A | #N/A | 1.3882341 | #N/A | #N/A | #N/A | Calcium-binding EF-hand family protein |
| BnaC09g52610D | at3g25600 | #N/A | -3.100569 | #N/A | #N/A | -1.492144 | #N/A | Calcium-binding EF-hand family protein |
| BnaA09g19500D | at3g25600 | #N/A | #N/A | 1.2178411 | 1.7300202 | #N/A | #N/A | Calcium-binding EF-hand family protein |
| BnaCnng21080D | at3g25600 | 1.8357303 | 1.1880562 | 2.9580374 | 2.8929281 | 1.7985562 | 1.780314 | Calcium-binding EF-hand family protein |
| BnaCnng49330D | at3g29000 | #N/A | #N/A | #N/A | #N/A | #N/A | -2.940749 | Calcium-binding EF-hand family protein |
| BnaC06g12630D | at3g29310 | -1.446082 | -2.502608 | #N/A | -1.100932 | -1.272518 | -1.420815 | calmodulin-binding protein-related |
| BnaA02g29820D | at3g29310 | #N/A | -2.470328 | #N/A | -1.482514 | #N/A | -1.701365 | calmodulin-binding protein-related |
| BnaA06g20230D | at3g43810 | #N/A | #N/A | -1.432438 | #N/A | #N/A | #N/A | EF hand domain protein encodes a calmodulin |
| BnaA03g08250D | at3g43810 | #N/A | #N/A | #N/A | -1.032432 | -1.447005 | #N/A | EF hand domain protein encodes a calmodulin |
| BnaA07g13000D | at3g43810 | #N/A | #N/A | #N/A | #N/A | -1.19418 | #N/A | EF hand domain protein encodes a calmodulin |
| BnaC03g10500D | at3g43810 | #N/A | #N/A | #N/A | #N/A | -1.143145 | #N/A | EF hand domain protein encodes a calmodulin |
| BnaC04g54820D | at3g43810 | #N/A | -1.839203 | -1.459987 | #N/A | #N/A | #N/A | EF hand domain protein encodes a calmodulin |
| BnaA06g20570D | at3g47480 | #N/A | #N/A | #N/A | -3.666992 | -5.684728 | -6.404409 | Calcium-binding EF-hand family protein |
| BnaCnng15180D | at3g47480 | #N/A | #N/A | #N/A | -3.188297 | -3.399273 | #N/A | Calcium-binding EF-hand family protein |
| BnaC08g20710D | at3g49050 | #N/A | #N/A | #N/A | -1.158064 | #N/A | -1.42744 | alpha/beta-Hydrolases superfamily protein |
| BnaA06g16240D | at3g49050 | #N/A | -2.296891 | #N/A | -1.349041 | #N/A | -1.126552 | alpha/beta-Hydrolases superfamily protein |
| BnaA06g20970D | at3g49260 | #N/A | 2.3104444 | 1.3972595 | 1.4229417 | 2.2515082 | 1.3497943 | IQ-domain 21 iqd21 |
| BnaC03g52530D | at3g49260 | #N/A | #N/A | #N/A | #N/A | 1.4888711 | 1.1243268 | IQ-domain 21 iqd21 |
| BnaA06g16060D | at3g49260 | 1.8078958 | #N/A | 1.7754605 | #N/A | 1.9338173 | #N/A | IQ-domain 21 iqd21 |
| BnaC08g20940D | at3g49260 | #N/A | #N/A | 2.6778276 | 2.1311533 | 4.8339667 | #N/A | IQ-domain 21 iqd21 |
| BnaA03g41500D | at3g51850 | #N/A | #N/A | #N/A | -1.460937 | #N/A | #N/A | member of Calcium Dependent Protein Kinase |
| BnaC08g23190D | at3g51850 | -4.415757 | #N/A | -2.048526 | -2.274054 | #N/A | -1.596831 | member of Calcium Dependent Protein Kinase |
| BnaC07g32550D | at3g51850 | -1.202996 | #N/A | -1.46656 | -1.982814 | -1.149012 | -2.234505 | member of Calcium Dependent Protein Kinase |
| BnaA09g32390D | at3g51850 | -1.939953 | -1.757135 | -1.215893 | -1.213949 | #N/A | -1.249213 | member of Calcium Dependent Protein Kinase |
| BnaC04g28550D | at3g51850 | #N/A | #N/A | #N/A | -1.083946 | #N/A | #N/A | member of Calcium Dependent Protein Kinase |
| BnaA04g05850D | at3g51920 | -1.625238 | #N/A | #N/A | -1.828939 | -1.99345 | #N/A | encodes a divergent member of calmodulin |
| BnaC04g28440D | at3g51920 | -1.156402 | -1.00549 | #N/A | #N/A | -1.723146 | #N/A | encodes a divergent member of calmodulin |
| BnaC04g28300D | at3g52290 | #N/A | -2.451141 | #N/A | -1.868109 | #N/A | #N/A | IQ-domain 3 IQD3 |
| BnaA04g05570D | at3g52290 | #N/A | #N/A | #N/A | -1.537866 | #N/A | #N/A | IQ-domain 3 IQD3 |
| BnaC06g14160D | at3g52870 | 1.8304652 | 2.128548 | 1.9257032 | 2.4456 | #N/A | 2.0768976 | IQ calmodulin-binding motif family protein |
| BnaA07g15810D | at3g52870 | #N/A | #N/A | #N/A | 1.745531 | #N/A | 1.2627362 | IQ calmodulin-binding motif family protein |
| BnaC06g14150D | at3g52870 | 1.698611 | #N/A | #N/A | 2.7663932 | #N/A | 2.4148352 | IQ calmodulin-binding motif family protein |
| BnaA04g05160D | at3g52870 | #N/A | 7.1611657 | 1.0588799 | #N/A | #N/A | #N/A | IQ calmodulin-binding motif family protein |
| BnaC04g27770D | at3g52870 | 1.2425846 | #N/A | #N/A | #N/A | #N/A | 1.3173321 | IQ calmodulin-binding motif family protein |
| BnaAnng14400D | at3g56690 | 1.7798721 | 2.021967 | 1.8740809 | 2.531407 | 1.9818414 | 1.9347467 | encodes a protein similar to ATPases and binds to calmodulin in vitro |
| BnaC08g27560D | at3g56690 | 1.7060088 | 1.4068724 | 1.2580366 | 1.4185544 | 1.0089508 | 1.5575228 | encodes a protein similar to ATPases and binds to calmodulin in vitro |
| BnaC04g24880D | at3g56760 | -1.037522 | #N/A | #N/A | #N/A | #N/A | -1.554183 | Protein kinase superfamily protein |
| BnaA04g02760D | at3g56800 | -1.35762 | -1.588902 | -1.005732 | #N/A | -1.844091 | #N/A | encodes a calmodulin |
| BnaA09g36050D | at3g56800 | -1.219097 | #N/A | -1.238159 | #N/A | #N/A | #N/A | encodes a calmodulin |
| BnaC03g23130D | at3g56800 | #N/A | 1.3710102 | 2.8440983 | 3.126689 | #N/A | #N/A | encodes a calmodulin |
| BnaC04g24860D | at3g56800 | #N/A | -2.168115 | #N/A | #N/A | -1.66491 | #N/A | encodes a calmodulin |
| BnaCnng20380D | at3g56800 | #N/A | #N/A | #N/A | #N/A | -1.164491 | #N/A | encodes a calmodulin |
| BnaCnng07470D | at3g57330 | -1.239265 | -1.036336 | #N/A | #N/A | #N/A | #N/A | autoinhibited Ca2+-ATPase 11 ACA11 |
| BnaC08g28250D | at3g57330 | 2.9731278 | 2.260514 | 3.523899 | #N/A | #N/A | #N/A | autoinhibited Ca2+-ATPase 11 ACA11 |
| BnaA09g36660D | at3g57330 | #N/A | #N/A | 1.7458506 | #N/A | #N/A | #N/A | autoinhibited Ca2+-ATPase 11 ACA11 |
| BnaCnng07350D | at3g57530 | #N/A | #N/A | 1.039165 | #N/A | #N/A | #N/A | Calcium-dependent Protein Kinase. |
| BnaA07g17350D | at3g57530 | #N/A | 1.3267003 | #N/A | #N/A | #N/A | #N/A | Calcium-dependent Protein Kinase. |
| BnaC08g29930D | at3g59690 | #N/A | #N/A | #N/A | -2.685007 | -3.318491 | -4.03288 | IQ-domain 13 IQD13 |
| BnaA09g37960D | at3g59690 | #N/A | #N/A | #N/A | #N/A | -1.911833 | -6.223252 | IQ-domain 13 IQD13 |
| BnaC04g22650D | at3g59820 | 3.1008883 | 2.892808 | 2.7659764 | 2.883 | 3.250417 | 2.700602 | LETM1-like protein |
| BnaC04g21980D | at3g61050 | #N/A | #N/A | #N/A | #N/A | #N/A | 1.0726007 | NTMC2T4 |
| BnaA04g00920D | at3g61050 | #N/A | 1.4737849 | #N/A | #N/A | #N/A | #N/A | NTMC2T4 |
| BnaCnng13400D | at3g61050 | #N/A | 1.3805625 | 1.5017182 | 1.1756592 | #N/A | 1.3558828 | NTMC2T4 |
| BnaC03g32080D | at4g00500 | -1.433725 | #N/A | -1.72677 | -1.756731 | #N/A | -2.001437 | alpha/beta-Hydrolases superfamily protein |
| BnaA03g27100D | at4g00500 | -2.594842 | #N/A | -2.909241 | #N/A | #N/A | #N/A | alpha/beta-Hydrolases superfamily protein |
| BnaC03g31610D | at4g00820 | #N/A | #N/A | 1.5837729 | #N/A | #N/A | #N/A | IQ-domain 17 iqd17 |
| BnaA02g21060D | at4g04720 | #N/A | #N/A | 1.230942 | #N/A | #N/A | #N/A | member of Calcium Dependent Protein Kinase |
| BnaC09g23000D | at4g05520 | 2.53133 | #N/A | 2.8457904 | #N/A | #N/A | #N/A | Encodes AtEHD2, one of the Arabidopsis Eps15 homology domain proteins involved in endocytosis AtEHD1, At3g20290 |
| BnaA03g24050D | at4g09570 | 1.1542126 | 1.672552 | 1.3155271 | #N/A | #N/A | #N/A | Encodes a member of Calcium Dependent Protein Kinase CDPK |
| BnaC03g28620D | at4g09570 | 1.0726246 | 1.6189828 | #N/A | #N/A | #N/A | #N/A | Encodes a member of Calcium Dependent Protein Kinase CDPK |
| BnaA01g10690D | at4g20780 | -1.431552 | #N/A | #N/A | #N/A | -1.58312 | -1.35953 | Calcium sensor involved in trichome branching. |
| BnaC07g36400D | at4g20780 | 2.824788 | 2.8284743 | 3.4722292 | 3.2200131 | 2.9592195 | 2.6635027 | Calcium sensor involved in trichome branching. |
| BnaA01g11650D | at4g21820 | 6.0373254 | #N/A | #N/A | #N/A | #N/A | #N/A | calmodulin binding |
| BnaC01g13360D | at4g21940 | 1.7124324 | #N/A | #N/A | #N/A | #N/A | #N/A | member of Calcium Dependent Protein Kinase |
| BnaC01g41430D | at4g23060 | #N/A | #N/A | #N/A | 2.1096604 | #N/A | 1.4464114 | IQ-domain 22 IQD22 |
| BnaA03g46140D | at4g23650 | #N/A | #N/A | 1.0235826 | #N/A | #N/A | #N/A | Encodes calcium dependent protein kinase 3 CPK3 |
| BnaA01g13330D | at4g23650 | #N/A | #N/A | #N/A | #N/A | -1.072311 | #N/A | Encodes calcium dependent protein kinase 3 CPK3 |
| BnaC07g50270D | at4g23650 | #N/A | #N/A | 1.096103 | 1.2762738 | #N/A | #N/A | Encodes calcium dependent protein kinase 3 CPK3 |
| BnaA01g14990D | at4g25800 | #N/A | #N/A | #N/A | #N/A | #N/A | 1.1397066 | Calmodulin-binding protein |
| BnaC01g18690D | at4g26570 | 1.1625011 | #N/A | #N/A | #N/A | #N/A | #N/A | member of AtCBLs Calcineurin B-like Calcium Sensor Proteins |
| BnaA10g08320D | at4g27280 | 1.2225536 | 1.186068 | 1.0764055 | #N/A | #N/A | #N/A | Calcium-binding EF-hand family protein |
| BnaCnng38440D | at4g27280 | 2.5229616 | #N/A | 1.6518553 | #N/A | #N/A | #N/A | Calcium-binding EF-hand family protein |
| BnaC01g19450D | at4g27280 | 4.281868 | #N/A | 4.320066 | 1.8172219 | 1.7408322 | 1.3513324 | Calcium-binding EF-hand family protein |
| BnaA06g19640D | at4g28600 | #N/A | #N/A | 1.474534 | #N/A | #N/A | #N/A | encodes a calmodulin-binding protein |
| BnaC01g08580D | at4g29900 | -1.287565 | #N/A | #N/A | -1.453579 | #N/A | #N/A | one of the type IIB calcium pump isoforms |
| BnaA01g07100D | at4g29900 | -1.454176 | #N/A | #N/A | #N/A | -1.062568 | #N/A | one of the type IIB calcium pump isoforms |
| BnaA03g49860D | at4g29900 | -1.207201 | #N/A | #N/A | #N/A | #N/A | #N/A | one of the type IIB calcium pump isoforms |
| BnaC07g42210D | at4g29900 | -1.06299 | #N/A | #N/A | -1.180434 | #N/A | #N/A | one of the type IIB calcium pump isoforms |
| BnaA01g06120D | at4g31000 | -6.531093 | -4.082888 | #N/A | -3.901425 | -5.558741 | -3.67118 | Calmodulin-binding protein |
| BnaA01g04850D | at4g32060 | #N/A | #N/A | -1.183496 | #N/A | -1.439195 | #N/A | calcium-binding EF hand family protein |
| BnaC01g06390D | at4g32060 | #N/A | #N/A | #N/A | #N/A | -1.377846 | #N/A | calcium-binding EF hand family protein |
| BnaA01g03820D | at4g33050 | #N/A | #N/A | #N/A | #N/A | -1.970185 | -4.794896 | embryo sac development arrest 39 EDA39 |
| BnaC03g66780D | at4g33050 | 1.3471143 | 1.1902685 | #N/A | #N/A | 1.4576812 | #N/A | embryo sac development arrest 39 EDA39 |
| BnaA03g50900D | at4g34150 | #N/A | #N/A | 1.1875778 | #N/A | #N/A | #N/A | Calcium-dependent lipid-binding CaLB domain |
| BnaA08g11360D | at4g34150 | #N/A | 1.1544995 | #N/A | #N/A | #N/A | #N/A | Calcium-dependent lipid-binding CaLB domain |
| BnaC03g77080D | at4g34150 | 1.0405236 | 1.3378915 | 1.3490882 | #N/A | #N/A | #N/A | Calcium-dependent lipid-binding CaLB domain |
| BnaA03g53250D | at4g35310 | #N/A | #N/A | #N/A | -5.51686 | #N/A | -4.898857 | calmodulin-domain protein kinase CDPK isoform 5 CPK5 |
| BnaA08g15730D | at4g37640 | -2.857732 | -6.826244 | -4.945241 | #N/A | #N/A | #N/A | Encodes a calmodulin-regulated Ca2+-pump |
| BnaC03g61330D | at4g37640 | -2.161327 | -2.069555 | -2.315683 | #N/A | -1.661673 | -2.532146 | Encodes a calmodulin-regulated Ca2+-pump |
| BnaA03g54160D | at4g37640 | 1.872424 | #N/A | #N/A | #N/A | #N/A | #N/A | Encodes a calmodulin-regulated Ca2+-pump |
| BnaC07g47590D | at4g38810 | 1.4680347 | 1.5839336 | 1.9951025 | 2.4812677 | 1.6788256 | 2.5942638 | Calcium-binding EF-hand family protein |
| BnaA06g37340D | at4g38810 | 2.921965 | 2.6110308 | 2.59173 | 3.822338 | 2.6508443 | 3.5868325 | Calcium-binding EF-hand family protein |
| BnaA03g00740D | at5g03040 | #N/A | #N/A | #N/A | -1.1682 | #N/A | #N/A | IQ-domain 2 iqd2 |
| BnaA10g26820D | at5g03040 | -2.256249 | -2.032551 | #N/A | -1.118695 | #N/A | #N/A | IQ-domain 2 iqd2 |
| BnaC03g01030D | at5g03040 | -4.654191 | -2.277748 | #N/A | -1.180775 | -1.570791 | -1.235416 | IQ-domain 2 iqd2 |
| BnaA10g26150D | at5g04020 | -1.874604 | #N/A | #N/A | -1.847978 | -2.188509 | -1.764147 | calmodulin binding |
| BnaCnng10300D | at5g04170 | -1.313829 | -1.067467 | -1.055062 | -1.314449 | -1.080141 | -1.289411 | Calcium-binding EF-hand family protein |
| BnaC05g28220D | at5g04220 | 1.1719002 | #N/A | #N/A | 1.2355002 | 1.0595555 | 2.3910952 | SYTC |
| BnaCnng10250D | at5g04220 | #N/A | #N/A | 1.7155066 | 1.2467808 | #N/A | 1.55381 | SYTC |
| BnaA10g25990D | at5g04220 | #N/A | 1.0099789 | #N/A | #N/A | #N/A | #N/A | SYTC |
| BnaA10g25410D | at5g04870 | #N/A | #N/A | 1.1798152 | #N/A | #N/A | #N/A | A calcium-dependent protein kinase |
| BnaC09g50440D | at5g04870 | #N/A | #N/A | 1.0397717 | #N/A | #N/A | #N/A | A calcium-dependent protein kinase |
| BnaC03g02960D | at5g07240 | -1.650397 | #N/A | #N/A | #N/A | #N/A | #N/A | IQ-domain 24 IQD24 |
| BnaA03g01970D | at5g07240 | #N/A | #N/A | #N/A | #N/A | #N/A | -1.226575 | IQ-domain 24 IQD24 |
| BnaA10g23750D | at5g07300 | 6.417388 | 2.325832 | 5.4390607 | 4.854026 | 4.3216453 | 5.6653004 | Encodes a copine-like protein |
| BnaC09g48510D | at5g07300 | 3.708408 | 2.179671 | 2.4620216 | 2.9997382 | 2.9968817 | 2.8991928 | Encodes a copine-like protein |
| BnaC05g10710D | at5g07300 | #N/A | #N/A | 5.624918 | #N/A | #N/A | #N/A | Encodes a copine-like protein |
| BnaA03g15220D | at5g07300 | #N/A | -4.580647 | #N/A | -2.199377 | #N/A | #N/A | Encodes a copine-like protein |
| BnaC09g48520D | at5g07300 | 6.3825593 | #N/A | 3.269725 | #N/A | #N/A | #N/A | Encodes a copine-like protein |
| BnaC03g02980D | at5g07300 | #N/A | #N/A | #N/A | 1.1693983 | #N/A | 1.805724 | Encodes a copine-like protein |
| BnaA03g01990D | at5g07300 | 1.1826775 | #N/A | 1.7226278 | 1.0226188 | 1.326992 | 1.2275285 | Encodes a copine-like protein |
| BnaA03g02570D | at5g08580 | #N/A | #N/A | #N/A | 1.1097819 | #N/A | #N/A | Calcium-binding EF hand family protein |
| BnaA10g22860D | at5g08580 | #N/A | #N/A | #N/A | #N/A | #N/A | -2.023802 | Calcium-binding EF hand family protein |
| BnaC09g24410D | at5g12180 | #N/A | #N/A | -3.199686 | -5.216468 | #N/A | #N/A | member of Calcium Dependent Protein Kinase |
| BnaA10g29980D | at5g12480 | #N/A | #N/A | 1.0961369 | #N/A | #N/A | #N/A | calmodulin-domain protein kinase CDPK isoform 7 CPK7 |
| BnaC02g08720D | at5g19450 | -1.265858 | #N/A | #N/A | -1.799906 | #N/A | -1.162456 | calcium-dependent protein kinase CDPK19 |
| BnaC02g41570D | at5g23580 | #N/A | #N/A | #N/A | -1.945944 | -1.028968 | -2.350952 | unique family of enzymes containing a putative calcium-binding EF hands structure at the carboxyl terminus |
| BnaA02g32820D | at5g23580 | -1.209349 | 3.1405878 | #N/A | -1.952231 | #N/A | -1.515624 | unique family of enzymes containing a putative calcium-binding EF hands structure at the carboxyl terminus |
| BnaC09g04790D | at5g23580 | #N/A | #N/A | -1.014983 | -1.327217 | #N/A | -1.560038 | unique family of enzymes containing a putative calcium-binding EF hands structure at the carboxyl terminus |
| BnaA09g05220D | at5g23580 | #N/A | #N/A | #N/A | #N/A | 1.3461452 | #N/A | unique family of enzymes containing a putative calcium-binding EF hands structure at the carboxyl terminus |
| BnaA06g26290D | at5g23580 | #N/A | #N/A | #N/A | -1.832254 | #N/A | #N/A | unique family of enzymes containing a putative calcium-binding EF hands structure at the carboxyl terminus |
| BnaC07g30190D | at5g24270 | #N/A | #N/A | 2.6866202 | 2.4943156 | #N/A | #N/A | encodes a calcium sensor |
| BnaC09g04430D | at5g24430 | -1.547102 | #N/A | #N/A | #N/A | -1.370601 | #N/A | Calcium-dependent protein kinase CDPK family protein |
| BnaA09g04890D | at5g24430 | -1.831351 | -1.192966 | #N/A | -1.106521 | -1.447575 | #N/A | Calcium-dependent protein kinase CDPK family protein |
| BnaA09g04040D | at5g26920 | #N/A | #N/A | -1.120054 | -1.272908 | -2.081906 | -1.178409 | Encodes a calmodulin-binding protein CBP60g |
| BnaC09g03400D | at5g26920 | #N/A | #N/A | #N/A | -1.714331 | -2.670111 | -2.159174 | Encodes a calmodulin-binding protein CBP60g |
| BnaAnng28220D | at5g26920 | 2.9759233 | 2.5284712 | 2.0023181 | 1.0097163 | #N/A | #N/A | Encodes a calmodulin-binding protein CBP60g |
| BnaC07g28420D | at5g26920 | 2.2982256 | 2.1116307 | 1.4048783 | 1.266222 | #N/A | #N/A | Encodes a calmodulin-binding protein CBP60g |
| BnaA09g03510D | at5g28850 | #N/A | #N/A | #N/A | #N/A | #N/A | 1.3707819 | Calcium-binding EF-hand family protein |
| BnaC09g02810D | at5g28850 | 1.3922161 | #N/A | #N/A | 1.3496771 | #N/A | #N/A | Calcium-binding EF-hand family protein |
| BnaCnng38590D | at5g35670 | #N/A | #N/A | #N/A | -2.547183 | #N/A | #N/A | IQ-domain 33 iqd33 |
| BnaC04g30400D | at5g37710 | #N/A | #N/A | 2.4003701 | 1.0156995 | #N/A | 1.2513001 | alpha/beta-Hydrolases superfamily protein |
| BnaC06g43540D | at5g37770 | #N/A | #N/A | 1.2615374 | 1.7312111 | #N/A | 1.3179412 | calmodulin |
| BnaA07g25810D | at5g37770 | 1.2750083 | #N/A | 2.095109 | 1.8956933 | 1.7666323 | #N/A | calmodulin |
| Bnaunng00010D | at5g37780 | 2.3561196 | 5.4598103 | 3.147127 | 2.6122262 | 2.8682084 | 2.4960332 | encodes a calmodulin |
| BnaC04g34470D | at5g37780 | 2.5569885 | 2.1764243 | 2.7182615 | 3.0102594 | 2.2669904 | 2.9406908 | encodes a calmodulin |
| BnaC06g43530D | at5g37780 | 1.7351379 | 1.185575 | 1.6816806 | 1.2582711 | 1.535301 | 1.134277 | encodes a calmodulin |
| BnaA07g25830D | at5g37780 | #N/A | 1.6984528 | 1.1620299 | #N/A | #N/A | #N/A | encodes a calmodulin |
| BnaC04g31420D | at5g39380 | -2.405681 | #N/A | #N/A | -1.149553 | #N/A | #N/A | Plant calmodulin-binding protein-related |
| BnaA04g09200D | at5g39380 | -1.274971 | #N/A | #N/A | #N/A | #N/A | #N/A | Plant calmodulin-binding protein-related |
| BnaC07g24980D | at5g40190 | -1.032928 | -1.478132 | -2.104907 | #N/A | -1.357951 | #N/A | calmodulin-binding proteins |
| BnaC09g02710D | at5g49480 | #N/A | #N/A | #N/A | 1.424164 | #N/A | 1.4397231 | AtCP1 encodes a novel Ca2+-binding protein |
| BnaA09g03360D | at5g49480 | #N/A | 1.0062526 | 2.3925493 | 2.7335174 | #N/A | 2.2961612 | AtCP1 encodes a novel Ca2+-binding protein |
| BnaC02g13790D | at5g54130 | -1.711847 | #N/A | -1.472873 | -1.153781 | #N/A | #N/A | Calcium-binding endonuclease/exonuclease/phosphatase family |
| BnaCnng16590D | at5g54490 | 1.3663701 | #N/A | 2.5348272 | 1.5597148 | #N/A | #N/A | Encodes a PINOID PID |
| BnaA02g09590D | at5g54490 | 2.3333902 | 2.1150672 | 4.0755816 | 2.454452 | #N/A | #N/A | Encodes a PINOID PID |
| BnaA10g10600D | at5g56360 | #N/A | -1.463641 | #N/A | -1.130548 | #N/A | #N/A | Encodes PSL4, beat-subunit of endoplasmic reticulum-resident glucosidase II |
| BnaCnng14010D | at5g56360 | -1.305255 | -1.790996 | #N/A | -1.154989 | #N/A | -1.152734 | Encodes PSL4, beat-subunit of endoplasmic reticulum-resident glucosidase II |
| BnaC03g13570D | at5g56360 | #N/A | -1.080528 | #N/A | -1.656978 | #N/A | -1.43554 | Encodes PSL4, beat-subunit of endoplasmic reticulum-resident glucosidase II |
| BnaC01g41570D | at5g57110 | 2.0900512 | #N/A | #N/A | -1.502184 | #N/A | #N/A | Arabidopsis-autoinhibited Ca2+ -ATPase |
| BnaC01g17950D | at5g57110 | #N/A | #N/A | #N/A | #N/A | 1.0213968 | #N/A | Arabidopsis-autoinhibited Ca2+ -ATPase |
| BnaA10g11510D | at5g57580 | #N/A | 1.4866099 | 1.4382826 | #N/A | #N/A | 1.2239622 | Calmodulin-binding protein |
| BnaCnng63910D | at5g57580 | #N/A | 1.7767723 | 1.0198019 | #N/A | #N/A | #N/A | Calmodulin-binding protein |
| BnaC02g11430D | at5g57580 | #N/A | #N/A | #N/A | -1.577347 | -1.663849 | -1.896276 | Calmodulin-binding protein |
| BnaA02g35270D | at5g57580 | #N/A | #N/A | -1.303499 | -1.061672 | -2.145488 | -1.46802 | Calmodulin-binding protein |
| BnaAnng07030D | at5g61790 | #N/A | #N/A | 1.398088 | #N/A | #N/A | #N/A | calnexin 1 CNX1 |
| BnaC09g05410D | at5g61790 | #N/A | 1.2871753 | #N/A | #N/A | #N/A | #N/A | calnexin 1 CNX1 |
| BnaC03g52250D | at5g61790 | #N/A | #N/A | #N/A | #N/A | -1.540442 | #N/A | calnexin 1 CNX1 |
| BnaA09g05810D | at5g61790 | 1.0114595 | #N/A | #N/A | #N/A | -1.142639 | #N/A | calnexin 1 CNX1 |
| BnaA06g21300D | at5g61790 | #N/A | -1.177755 | #N/A | -1.017868 | -1.694571 | #N/A | calnexin 1 CNX1 |
| BnaA06g21540D | at5g62070 | 3.2575264 | #N/A | 2.0013847 | 2.2534795 | 2.120372 | #N/A | IQ-domain 23 IQD23 |
| BnaC03g51980D | at5g62070 | 2.9357662 | #N/A | 3.0006447 | 1.2759426 | 2.204024 | 1.6371902 | IQ-domain 23 IQD23 |
| BnaC03g74970D | at5g62390 | -2.340892 | -3.273902 | -3.105012 | -1.649305 | #N/A | #N/A | A member of Arabidopsis BAG Bcl-2-associated athanogene |
| BnaCnng30830D | at5g62570 | #N/A | #N/A | 1.5528167 | #N/A | #N/A | #N/A | Calmodulin binding protein-like |
| BnaA06g22060D | at5g62570 | #N/A | 1.2421597 | 1.1928079 | #N/A | #N/A | #N/A | Calmodulin binding protein-like |
| BnaAnng36110D | at5g66210 | 1.0879397 | #N/A | #N/A | #N/A | #N/A | #N/A | member of Calcium Dependent Protein Kinase |
| BnaC07g16820D | at5g66210 | #N/A | #N/A | #N/A | #N/A | #N/A | -1.767124 | member of Calcium Dependent Protein Kinase |
| BnaC09g08180D | at5g66210 | #N/A | #N/A | #N/A | #N/A | -1.195814 | -1.042537 | member of Calcium Dependent Protein Kinase |
| BnaA07g12770D | at5g66210 | #N/A | #N/A | #N/A | #N/A | #N/A | 1.096709 | member of Calcium Dependent Protein Kinase |

# Table S4 CBL1 genes of two cultivars in RNA-seq

T1: the tolerant cultivar C18 under 2°C compared to 22°C; T2: the tolerant cultivar C18 under 0°C compared to 22°C; T3: the tolerant cultivar C18 under -2°C compared to 22°C; S1: the sensitive cultivar C20 under 2°C compared to 22°C; S2: the sensitive cultivar C20 under 0°C compared to 22°C; S3: the sensitive cultivar C20 under -2°C compared to 22°C

| Gene | Tair_gene | tair_anno | Log2(S1) | Log2(S2) | Log2(S3) | Log2(T1) | Log2(T2) | Log2(T3) |
| --- | --- | --- | --- | --- | --- | --- | --- | --- |
| BnaA01g08510D | AT4G17615 | CBL1 | 0.00 | 0.00 | 4.23 | 0.00 | 0.00 | -2.60 |
| BnaA03g43160D |  |  | 0.00 | 0.00 | 1.74 | 0.00 | 0.00 | 0.00 |
| BnaC01g10210D |  |  | 1.57 | 0.00 | 4.11 | 0.00 | 0.00 | 0.00 |
| BnaC07g34450D |  |  | 0.00 | 0.00 | 1.92 | 0.00 | -2.14 | 0.00 |

| Table S5 The primers used to qRT-PCR in this study | | | |
| --- | --- | --- | --- |
| gene name | Forward primer(5'-3') | Reverse primer(5'-3') | description |
| BnaC09g10170D | ATGGGTAAAGTGGCTGTTGGT | TCAGCTTCCCTATCGGCG | BnHXK-2 |
| BnaA09g10080D | ATGGGTAAAGTGGCTGTTGGT | GCTTCCCTATCGGCGTCG | BnHXK-2 |
| BnaC05g26650D | GATTGTACATGAACTACACCATGTTTA | TTATGACTGTAAAGAGGCAACGA | BnHXK-3 |
| BnaA05g15840D | AAGAGGCTTTGGTGGAGATTTTA | CATGACTGTAAAGAGGCAACGAG | BnHXK-3 |
| BnaC08g35240D | AGTTTCATTCTTTCCACCCAGT | ATGTTCTCCAGCATAGCCCTC | BnMIPS 2 |
| BnaA09g42760D | CATCGAGAGCTTCAAGGTAGAG | CGACCCATTGGTAAGTACCGTT | BnMIPS 2 |
| BnaC04g34650D | GCAGAGGGCTATGCTGGAA | TCTTCATCCTCCCTGTCCTTC | BnMIPS 2 |
| BnaC03g50280D | TACCGTGAAAAGATTTGGGAC | TAGCAACGATGATGCCAGTGT | BnIMP |
| BnaC09g06200D | AGCCAAGCGAAGTATGGAGCA | CGATCCCACCAGCCTCTGTAA | BnIMP |
| BnaC09g10140D | TACACACACTTGATGAGCGAAGAA | TCACCATTTTAGTTTTGCCGG | BnMIOX 2 |
| BnaA09g10050D | AGTGAAGAAGACAAGGAAGATCTCAA | TCACCATTTTAGTTTTGCCGG | BnMIOX 2 |
| BnaA07g00980D | TGAACGATGAAGACAGGGAGG | TCACCATTTTAGTTTTGCCGG | BnMIOX 2 |
| BnaC07g40160D | ATGACAATCTCTATTGAGAAGCCGA | GCCTCTCATTCTCAGCATCGTAGT | BnMIOX 4 |
| BnaA03g47910D | ATGACAATCTCTATTGAGAAGCCGA | TCTCATTCTCAGCATCGTAGTTCCT | BnMIOX 4 |
| BnaC07g39680D | CACCCAGTTTACAGAGGAGTTCG | ATCTCGGCTGTTAGGAAAGTACC | BnCBF1-1 |
| BnaAnng34260D | CGATGTGTTTTATATGGACGAGG | GCATCTCCGTCAAAGTCATAGTT | BnCBF1-2 |
| BnaA08g30930D | TTCTGAACTGCTCTGCTCCGA | CCTCACAGCAGGTTTCTTGG | BnCBF1-3 |
| BnaA08g30950D | CAATGAACACATTCCCTGCG | GGGATAATAATCACCTCCTGCTA | BnCBF1-4 |
| BnaA03g13620D | TCTGAAATGTTGGGCTCCGA | CGCGTCTCCCGAAACTTCTT | BnCBF2-1 |
| BnaA08g30910D | TGGTGATTACTGTCCCATGTTG | TCTTGTTTGGTTCCCTCACTTC | BnCBF2-2 |
| BnaA10g07630D | TTTCTCAGACTCGTTCCTCTCG | CTCCCTGCTCGTTTCTTCG | BnCBF4-1 |
| BnaC09g28190D | GACGGCTACGACTGAGACGA | CGGCCATATAAAACACACCG | BnCBF4-2 |
| BnaC01g20220D | TGGAAATTTATGAACTCCGCCA | CAGTAAATGGAACACGCATAGCC | BnCOLD1 |
| HM187577.1 | GGTCACAGCGAAAAACCAGAT | TCTTGGCGTGATAACCTGGAA | BnCOR25 |
| U14665.1 | TCTCATTGGGATTGGTTCTTCTTT | ATGTTGCCGTCACCTTTATCG | BnCOR15 |
| BnaA02g02910D | GGAGAAGGGTAATGTGCTGATGG | GCTACTTGTTCATGCCGGTCTT | BnCOR6.6 |
| BnaC07g34450D | GAGTGGAGCGATTTCGTGAAC | TCATTTGACAATCTCATCCACCT | BnCBL1-1 |
| BnaA01g08510D | TGGGCTGCTTCCACTCAA | CATCATCAACAACCGAACTGC | BnCBL1-2 |
| AF111812 | CTGGAATTGCTGACCGTATGAG | ATCTGTTGGAAAGTGCTGAGGG | BnActin |
